# The internal democracy of the crisis parties in Western Europe: a quantitative analysis of the role of digitalization, ideology and populism.

**DOI:** 10.12688/openreseurope.21115.1

**Published:** 2025-09-19

**Authors:** Jorge Bronet, Rosa Borge

**Affiliations:** 1 Communication Networks & Social Change research group (CNSC), Open University of Catalonia, Barcelona, Catalonia, 08018, Spain

**Keywords:** new political parties, intra-party democracy, participation, digitalization, populism, Western Europe.

## Abstract

**Background:**

Previous studies on the internal democracy of new digital parties in Western Europe suggest a plebiscitary tendency, but most focus on a limited number of cases. This paper aims to empirically analyze the intra-party democracy of electorally successful new parties in Western Europe and identify the main factors that may influence it.

**Methods:**

Drawing on data from the second round of the Political Parties Database (PPDB) and the first wave of the Populism and Political Parties Expert Survey (POPPA), this study covers more than 100 parties across 13 countries. Adopting a generational approach, we define a cohort of “crisis parties”—founded between the economic crisis and the pandemic—and examine their internal democracy in comparison to older parties, using Von dem Berge and Poguntke’s IPD model and Böhmelt *et al.*,’s (2022) framework, with ideology, digitalization, and populism treated as explanatory variables.

**Results:**

Our findings show that being a crisis party—even a highly digitalized one on the left—does not entail more plebiscitary forms of intra-party democracy.

**Conclusions:**

Digitalization emerges as the most consistent predictor shaping intra-party democracy, while the cohort effect matters only insofar as crisis parties are more populist than older parties, which ultimately reduces their internal democracy.

## Introduction

Over the last two decades, many new parties have emerged in Western Europe. The emergence of new parties is not a novelty, but their number and electoral success, especially in some countries, are particularly relevant during this period ( Chiaramonte & Emanuele, 2022). Apparently, some of these parties, at least in their first stage, have bet on increasing their internal democracy to reconnect with disaffected citizens by leaning on digital tools. Nevertheless, many authors point out that these parties are subject to electoral competition and become institutionalized, a fact that makes them take steps back in terms of internal democracy (for instance, Meloni & Lupato, 2023). Some scholars also claim that their participation model is essentially plebiscitary (the what, how, and when of what is discussed or asked remain in the hands of an elite), which triggers the effect of reinforcing the power of leaders and failing to empower members and citizens ( Barberà & Rodríguez-Teruel, 2020; Biancalana, 2016; Gerbaudo, 2019; Invernizzi-Accetti & Wolkenstein, 2017; Rodríguez-Teruel & Barberà, 2018). However, other studies note that some of these parties are not becoming less internally democratic ( Deseriis, 2020; Gad, 2020). Moreover, the analysis of Von dem Berge and Poguntke (2017) shows that plebiscitary intra-party democracy (PIPD) appears to be a complement for parties with high assembly-based intra-party democracy (AIPD) and parties with high levels of PIPD and low levels of AIPD are extremely unusual.

What we can basically find now in the scientific literature is descriptive studies of few cases ( Correa *et al.*, 2021). Most new European parties’ IPD studies consider only take into account one or a few parties ( Biancalana, 2016; Borge & Santamarina, 2016; Deseriis, 2020; Gad, 2020; Gerbaudo, 2019; Gherghina & Stoiciu, 2020; Rodríguez-Teruel & Barberà, 2018; Vodová & Voda, 2020). Therefore, quantitative research is needed to collect new empirical evidence that allows us to obtain a global vision and contrast what these case studies are pointing out by considering a significant number of observations, comparing them, and trying to identify patterns, if any. Fortunately, data on some of these new parties are available in the Political Parties Database ( Poguntke *et al.*, 2022; Scarrow *et al.*, 2022b) round 2 and in the Populism and Political Parties Expert Survey dataset (POPPA database, Meijers & Zaslove, 2020; Meijers & Zaslove, 2021) that we can analyze.

This paper aims to contribute to the academic debate on what the internal democracy of Western Europe’s new political parties is like and what variables can explain it. Are these parties more democratic than the older parties? Is their IPD different? And why? We provide new empirical evidence to respond to these questions better. By leaning on Bronet and Borge’s generational approach ( 2024) and Von dem Berge and Poguntke’s (2017) IPD model we analyze 98 Western European parties from 13 countries, 29 new and 69 old parties from the Political Parties Database (PPDB). To confirm and complete our results, we also observe 109 Western European parties from 11 countries, 40 new and 69 traditional parties, by operationalizing the Populism and Political Parties Expert Survey dataset ( Meijers & Zaslove, 2020; Meijers & Zaslove, 2021).

The article is structured as follows. In the next section, we review and discuss the most relevant literature on the internal democracy of the new Western European parties. In section three, we set the theoretical framework on which we rely to delimit the phenomenon of the new Western European parties and measure intra-party democracy. In section four, we formulate our hypotheses regarding the internal democracy of these parties, taking into account different explanatory variables, such as cohort, intra-party democracy, ideology, digitalization, and populism. In section five, we describe our dataset and methodology. In section six, we provide the results of our analysis by empirically verifying our hypotheses. Finally, in the last section, we summarize our main findings and suggest a new complementary research line.

## Internal democracy in new parties of Western Europe: the existing evidence

Since the literature usually claims that left-wing parties tend to be more sensitive or inclined toward internal democracy, these parties have garnered most of the attention of internal democracy scientific research. Parties from the left are frequently seen as having members who want to control parliamentary groups ( Pettitt, 2012). As Sandri *et al.* (2025) reflect, left-wing parties tend to place greater emphasis on internal democracy as a party goal, whereas right-wing parties often emphasize hierarchical control. Ideological preferences on the left tend to favor more horizontal models in the linkage between parties, whereas on the right, preferences for conventional vertical configurations are more common. Poguntke *et al.* (2016) note that families related to the left-wing seem to be more internally democratic, and Scarrow *et al.* (2022a) argue that certain party families (social democrats and green) are more likely to offer plebiscitary voting rights. Other scholars have described how parties use digital technologies, depending on their ideology. For instance, Blasco (2021) pointed out how left-wing party leaders adopt new technologies at a higher rate because these new tools promote more online participation ( Blasco, 2021). Vaccari (2013) and Raniolo *et al.* (2021) note that left-wing parties facilitate more online interactions; that is, they use ICTs for participation, while right-wing parties do so only for communication purposes. Correa *et al.* (2021) observed that Online Participation Platforms have mostly been developed by left-wing parties, but interestingly pointed out some exceptions linked to new center-right parties.

In fact, some new Western European parties, mostly left-wing, have shown a clear will to involve the voice of society in their decision-making processes (the very reason for their formation), defense participation, decentralization of power, and transparency. They were formed with the aim of being truly democratic, as opposed to the established parties (which were criticized for being disconnected from the citizenry). Their emergence generated a great deal of enthusiasm among a section of citizens who wanted to become involved in politics, but broke away from the hierarchical structure of traditional parties. Examples of this sort of party are Podemos (Spain), Movimento 5 Stelle (Italy), and Alternativet (Denmark).

In these cases, we find much literature related to internal democracy, primarily case studies. And this research generally suggests to us that it seems that their initial aim for participation is now declining. Many authors have described how these new parties have fallen victims to the pressures of competition and the institutionalization process ( Bennet *et al.*, 2018; Gad, 2020; Invernizzi-Accetti & Wolkenstein, 2017; Lioy *et al.*, 2019). They point to the tension between two opposing forces as affecting these parties: on the one hand, the intention to increase participation and deliberation, and on the other hand, the need to centralize power to compete at the polls. Kitschelt (2006) described movement parties (which logically have a strong focus on participation) as transitional phenomena. For him, they were movements on their way to becoming a party.

Some scholars warn that the greater the efforts for participation, the more negative is the impact on electoral outcomes ( Fitzpatrick, 2018). The more policy-seeking a party is, the worse its electoral performance. Conversely, the more leader-centered a party is, the more successful it is in its vote-seeking strategy and the better it performs at polls. Electoral competition rewards hierarchical structures with high leader personalization, and thus, parties with a consumer strategy (an extreme form of the cartel party) promote oligarchic structures ( Löfgren & Smith, 2003). As Strom (1990, p. 577) points out, the decentralization of political decision-making is detrimental to the maximization of votes. The less power leaders have at a party, the less electorally efficient it is. Following the line of Bennett *et al.* (2018), by struggling between internal democracy and cartel strategies (to strengthen their leaders) in order to compete within the system, these parties neither have the type of organization that most benefits political competition (vertical), nor have they the form that their electorate and activists demand.

It should be remembered that one of the factors that considerably affects parties whose primary objective is intra-party democracy and causes important changes is size ( Harmel & Janda, 1994). The rapid growth of these new parties, from movements to institutionalized parties, may have led to a change in their primary objective: how can they aggregate and articulate so many diverse interests? Having suffered a decline in votes (see Podemos, Syriza, or Movimento 5 Stelle), they might have turned to maximizing public office (entering coalition governments), thereby modifying their initial strategy of fostering participation.

The challenge facing these parties is how to institutionalize their organizations without undermining their internal democracy, which is the basis of their existence and, therefore, their great electoral claim ( Lioy *et al.*, 2019). How can they share authority between members and leaders in a stable mannerway? How can they manage the tension between the demand for greater internal democracy and the need for central leadership?

Meloni and Garcia Lupato (2023), studying the Podemos political party in Spain, pointed out some relevant regressions in the evolution of digital innovations for intra-party democracy. There was only one citizen consultation, while Podemos was in government ^1^ (from January 2020 to November 2023) and some hard setbacks were observed (including the abandonment of Plaza Podemos ^2^). None of these setbacks have been due to technical problems but are the result of party decisions. The authors described the tension between the initial organization and the institutionalization process, and between the forces of interactivity and control (and personalization) that this movement digital party suffers. Curiously, as Podemos left the government and lost many seats, at least two consultations were held ^3^.

However, some young parties do not seem to follow this centralization trend. There are a few recent Western European exponents, such as Piratenpartei ( Deseriis, 2020) or Alternativet ( Gad, 2020), which show us that parties can even move from participation to deliberation, from a vote-centric model (too plebiscitary) to a talk-centric model (party as forum). Nonetheless, despite experiencing explosive success at birth, the electoral results of these parties are currently deteriorating, and they struggle to maintain the level of deliberation offered at the time of their emergence (good levels of equality, diversity, and transparency) without losing competitiveness.

On a different note, relevant scientific literature points out how the new Western European parties, mostly from the left, which seemingly offer citizens more participation than other parties, appear to give them little or no power ( Lioy *et al.*, 2019; Rodríguez-Teruel & Barberà, 2018). Their participation models are essentially top-down ( Gerbaudo, 2019). Bottom-up participation is irrelevant, except when they emerged. Members’ ability to influence the political agenda (i.e., policy decisions and internal deliberations) would be low. Many authors argue that these young parties’ internal democracies have largely adopted a form of aggregative (atomistic, where individuals remain separated from each other and interact only with the party, preventing them from acting together), plebiscitary, or reactive participation ( Guglielmo, 2021). The what, how, and when of what is discussed or asked remain in the hands of an elite, which reinforces the power of leaders and fails to empower members and citizens ( Barberà & Rodríguez-Teruel, 2020; Biancalana, 2016; Gerbaudo, 2019; Invernizzi-Accetti & Wolkenstein, 2017; Rodríguez-Teruel & Barberà, 2018). Party elites may implement plebiscitary methods of intra-party decision-making processes in order to reduce the power of activists and increase their own control of the grassroots by empowering docile and passive members ( Von dem Berge & Poguntke, 2017). The concept of pseudo-participation has also been introduced to determine whether these parties use digital participation tools as a strategy to give people (citizens and members) the feeling or impression that they can influence their decision-making processes ( Biancalana & Vittori, 2021). When studying relevant cases, the power of innovation tools appears to be mainly symbolic.

Moreover, in the case of populist parties (many new parties in Western Europe would respond to this logic), we could expect a notable plebiscitary or direct form of intra-party democracy ( Poguntke *et al.*, 2016). Instead, Böhmelt *et al.* (2022) found that, despite what we could expect from their ideational form (their story should lead to a more internal democracy, especially, plebiscitary), populist European parties are generally “undemocratic.” They observed a significant negative relationship between populism and internal democracy. The more populist a party is, the less internally democratic it is, and the more leader-centric it is. These authors also found that left-wing populist parties are more internally democratic than right-wing populist parties.

Nevertheless, regarding the plebiscitary condition of new political parties, Von dem Berge and Poguntke (2017) showed that parties with high levels of plebiscitary intra-party democracy (PIPD) tend to have high levels of assembly-based intra-party democracy (AIPD) as well, and parties with low AIPD and high PIPD scores are extremely unusual. They claimed that PIPD appears to be a complement for parties with high AIPD. However, Von dem Berge and Poguntke (2017) did not consider any of these new parties, since none of these parties were included in the first round of PPDB data collection (2010–2014) that they operationalized, although some of them had already been formed. Hence, their observation that parties with high PIPD tend to have high AIPD may not be appropriate for young parties, and this trend is changing in Western Europe: are they currently high in PIPD without a correspondingly high level of AIPD?

In any case, we need to remember that parties are constantly evolving, and it is possible that some parties may have fostered intra-party democracy at one point but no longer. Some authors, such as Rodríguez-Teruel and Barberà (2018), suggest that in a moment of time, in an initial stage, these parties were more prone to participation but later became more centralized and hierarchical through their process of institutionalization. From an evolutionary perspective, the changes that parties have undergone should be considered.

What about the internal democracy of new right-wing parties? Very few studies have been conducted on the internal organizational dynamics of these parties. Although, as mentioned above, Poguntke *et al.* (2016) noted that families related to the left-wing seem to be more internally democratic, we notice in their study that the far-right parties are so close to the left socialists in terms of assembly-based IPD and have even more plebiscitary IPD. This observation, based on quantitative analysis, appears to be supported by some new parties' case studies, such as Kamenova’s work ( 2021). She found that the Alternative für Deutschland (Germany) displays a high degree of internal participation in policy formation. Contrary to the absence of internal democratic mechanisms, scholars usually point out when it comes to populist right parties. Other exceptions to this rule, mentioned by her, are the cases of Sweden Democrats and Italian Lega Nord. Kamenova suggests that these parties can deploy deliberative practices to strengthen their connections with citizens.

Lastly, regarding the use of digital tools for participation, according to the latest research, new parties do not exhibit relevant differences in the way representatives interact with those they represent compared to traditional parties ( De Marco *et al.*, 2019). A recent study by Sandri *et al.* (2025) shows that the oldest parties seem to be more open to using online participation platforms for their decision-making processes. Bronet and Borge (2024) found that new parties are a bit more digitalized than older ones, but not exactly for increasing participation mechanisms but for mobilizing resources.

## Theoretical framework: the generational approach and modes of internal democracy

If we want to know the internal democracy of the new Western European parties, it is necessary to accurately determine which political parties we are referring to. Bronet and Borge (2024) recently provided a generational approach based on the contextual conditions when these young parties emerge (sociological, institutional, economic, and technological factors). Their work seeks to delimit the phenomenon of the boom of electorally successful new parties in Western Europe in recent years and empirically observe whether the fact that these new parties share a historical origin may entail that they have some common features. Bronet and Borge propose overcoming the term “new” (and “digital”) to refer to these parties and adopt the concept of crisis parties. They define them as:

*“... the political parties formed in Western Europe in the 21st century, mainly from 2008 (economic crisis) to 2020 (pandemic), that have won at least one seat in the national or state parliament in at least one election, regardless of their ideology (from the extreme right to the radical left), the political issues they underscore or their provenance (i.e., whether they are genuinely/organizationally new or renewed). To fight against traditional parties that have lost legitimacy and to reconnect with citizens, most such parties use digital tools”.* ( Bronet & Borge, 2024, p. 14–15)

This generational approach provides us with a good cohort to select units of analysis and make comparisons. Having our cohort, we must decide how to measure the internal democracy of crisis parties. We propose the use of von dem Berge and Poguntke’s model (2017). They designed a two-dimensional model to assess the quality of intra-party democracy, combining assembly-based intra-party democracy (AIPD) and plebiscitary intra-party democracy (PIPD), as the different modes of intra-party decision-making that the literature usually agrees with. AIPD and PIPD respond to different logics ( Brause & Poguntke, 2025b; Poguntke *et al.*, 2016), representative and direct democracy, respectively. AIPD occurs when discussion and decision (voting) occur together, and it measures the inclusiveness of decision-making inside parties based on deliberation within party bodies and assemblies. PIPD occurs when discussion and decision-making (voting) happen separately, and it measures whether all party members or even supporters have a final vote on party matters. They built AIPD and PIPD indices by selecting certain variables from the Political Parties Database (PPDB) and assigning them values. The PPDB is a cross-national initiative to establish and update an online public database as a source of key information on political party organizations. As Brause and Poguntke (2025b) have recently stated, the indices for AIPD and PIPD are suitable and robust tools for cross-national analysis of intra-party democracy.

Picking up the gauntlet from Bronet and Borge (2024), the latest available data from the Political Party Database Project (PPDB) and the Von dem Berge and Poguntke’s (2017) model of intra-party democracy represent a great opportunity to shed more light on how the internal democracy of the crisis parties is from all ideologies (right-wing ones as well, less studied), compare it to older cohorts, and try to find explanations related to some variables such as ideology or digitalization. Apart from being more digitalized, having fewer staff, and having more members than older Western European parties, internal democracy might be another common trait of the crisis parties ( Bronet & Borge, 2024).

The Populism and Political Parties Expert Survey (POPPA), another cross-national project, also includes an internal democracy measure for many European political parties. Although this measurement, based on experts’ opinions, does not distinguish between AIPD and PIPD, its operationalization relies on, among other studies, Poguntke *et al.*’s (2016) conceptual definition:

*“As defined in Poguntke *et al.* ( 2016, p. 670; see also Scarrow, 2015), intra-party democracy ‘maximizes the involvement of party members in the decisions that are central to a party’s political life, including program writing, and personnel selection and other intraorganizational decision-making’. Intra-democratic parties thus ‘are founded on principles of participation, competition, representation, responsiveness and transparency’ ( Rahat & Shapira, 2017, p. 88). What matters primarily is whether party members can influence decision making, internal debate is possible (and the party leadership does not rule this out) and procedures are inclusive of various factions and organizational layers within a party ( Meijers & Zaslove, 2021; Rahat & Shapira, 2017; Scarrow, 2015).”* ( Böhmelt *et al.*, 2022, pp. 1144)

Therefore, POPPA offers us a good indicator to contrast our results on intra-party democracy obtained from the PPDB and allows us to introduce populism as an explanatory variable as well.

At this point, we identified some limitations in our theoretical framework. First, those that a generational approach may entail. Bronet and Borge (2024) pointed out the difficulty in selecting the cut-off years to set a cohort or the awareness that there can be great differences within a given generation. Despite this, a generational perspective allows us to examine how members of a group can be shaped by similar contexts to better understand change. Second, the limitations are derived from Von dem Berge and Poguntke’s IPD model. This model is essentially based on what statutes say, so we must bear in mind the existing gap between the official story and the real one. These scholars consider that what constitutes IPD is inclusiveness in the sense of Scarrow’s definition ( 2005): how wide the circle of party decision-makers is. Thus, they basically measure how many people can make decisions and focus on the final vote. More people involved in the decision-making process does not necessarily mean better democracy. The manner in which the decisions are made is also relevant. Indicators, such as those recently observed by Martínek and Malý (2024) for the case study of the Czech Pirate Party, are valuable (number of collective decisions or consultations, turnout, percentage ranges for the winning option, or the elite preferred position). Notwithstanding, these detailed factors that could enhance Von dem Berge and Poguntke’s IPD model are usually analyzed in case studies, but not when considering a substantial number of parties. In any case, by considering some important variables for measuring IPD, their model proves to be a sound tool for conducting a quantitative analysis that allows us to compare a significant number of parties, at least at the normative level, and especially for checking the plebiscitary character of the crisis parties. Finally, regarding POPPA, we must consider that it can be difficult to accurately capture concepts such as intra-party democracy and populism using expert judgements ( Böhmelt *et al.*, 2022).

## Hypothesis

On the one hand, as we have noted before, many authors claim that the participation model of some of the new parties is essentially plebiscitary ( Barberà & Rodríguez-Teruel, 2020; Biancalana, 2016; Gerbaudo, 2019; Invernizzi-Accetti & Wolkenstein, 2017; Rodríguez-Teruel & Barberà, 2018). Moreover, in the case of populist parties (many of the crisis parties would respond to this logic), we could expect a very low AIPD score to correlate with a high PIPD ( Poguntke *et al.*, 2016).

On the other hand, Von dem Berge and Poguntke (2017) observe that plebiscitary intra-party democracy (PIPD) appears to be a complement for parties with high AIPD (assembly-based intra-party democracy) and parties with high levels of PIPD and low levels of AIPD are extremely unusual. Nevertheless, as we mentioned previously, Von dem Berge and Poguntke (2017) did not consider the new parties we are focusing on, since they were not included in the first round of PPDB data collection (2010–2014). Therefore, their observation that parties with high PIPD scores tend to have high AIPD scores may not be appropriate for crisis parties, especially if many of them are populists.

Thus, the first question we seek to clarify with our analysis is whether the internal democracy of the crisis parties is as plebiscitary as expected. The second important issue that we want to contribute more evidence is ideology. Is ideology a factor that influences the internal democracy of the crisis parties? As pointed out above, the literature usually claims that left-wing parties tend to be more sensitive or inclined toward internal democracy (see Pettitt, 2012; Poguntke *et al.*, 2016; or Sandri *et al.*, 2025). Nevertheless, some scholars stress how some new centrist ( Correa *et al.*, 2021) and right parties are showing signals of more internal democracy. For instance, that is the case of Alternative für Deutschland (a German far right party) that displays a high degree of internal participation ( Kamenova, 2021). Thus, it is important to first determine whether different internal democracy levels still exist in terms of ideology among crisis parties. Finally, if it is true that the crisis parties are or were more digitalized than the older parties ( Bronet & Borge, 2024) and technology makes it easier for parties to give all members a direct say, if they want to, by easing the voting process ( Sandri *et al.*, 2025, p. 6), we can expect a positive relationship between digitalization and plebiscitary IPD. Thus, we formulate our first hypothesis as follows:

*H1. The crisis parties are more plebiscitary than the older cohorts, especially if the crisis parties are on the left and highly digitalized. Just the contrary, older cohort’s parties, especially the old left-wing parties, have higher levels of assembly-based IPD than the crisis parties.*

Nonetheless, Böhmelt *et al.* (2022) found that, despite what we could expect from their ideational form (their story should lead to a more internal democracy, especially, plebiscitary), populist European parties are generally “undemocratic.” By operationalizing the POPPA, they observed a negative and significant relationship between populism and internal democracy. The more populist a party is, the less internally democratic it is and the more leader-centric it is. Hence, we could expect populist crisis parties to be less internally democratic. These authors also found that although this negative relationship prevails when considering only left-wing populist parties, these are more internally democratic than right-wing populist parties. Thus, our second hypothesis is as follows:

*H2. Populism is a factor that nuances the previous hypothesis in the sense that makes left-wing crisis parties that are populist to be less democratic than older left-wing parties, though are more democratic than the populist right ones.*

## Data and methods

Our dataset comes from two different sources, each related to one of our two hypotheses. We have gone to the Political Parties Database (PPDB) Round 2 to answer our first hypothesis (H1). Released in March 2022, the PPDB covers 288 parties in 51 countries, with 427 variables coded between 2016 and 2019. Following Bronet and Borge (2024), for our study we selected political parties from Western European countries ^4^ and considered as crisis parties those founded from the year 2006 onwards. PPDB Round 2 does not include any crisis parties from Finland, Sweden, and the United Kingdom; thus, we do not take into account the 23 older parties coded in our study to avoid biases, in line with Bronet and Borge (2024). Therefore, our sample comprised 29 crisis parties and 69 older cohort parties from 13 countries. A total of 98 Western European parties. See our shared dataset ( Bronet, 2025) to know which parties (crisis and older, respectively) are included in our study. For obtaining a brief description of some of the main features of the crisis parties PPDB sample, consult Bronet and Borge work ( 2024, pp. 18–19).

To conduct our analysis, we first take advantage of the variables created by Bronet and Borge (2024) and the data they coded for their work, namely, year of party foundation and cohort, region of Europe, left-center-right ideology, and digitalization index. We bet on the Bronet and Borge’s digitalization index because, in a very similar way as González-Cacheda and Cancela Outeda (2024), they have built it by taking into account 8 PPDB variables from the second round related to the parties’ websites affordances, the same database that we operate for analyzing the IPD indices. We are aware that there are some recent digitalization studies such as Sandri *et al.* (2025) that are more developed but the PPDB includes more countries and the coding dates are different. Second, we added the IPD indices. The IPD scores of the political parties were calculated following Von dem Berge and Poguntke’s IPD model ( 2017) and kindly provided to us for this research by Brause and Poguntke (2025a) when they were not yet published. Go to our shared dataset ( Bronet, 2025) for the characteristics of the new variables considered in this study.

With regard to this dataset, we identified some issues. The PPDB, which is based on the collaboration of expert coders, has some missing data ( Scarrow *et al.*, 2022b). It can be observed that many of the extra-parliamentary political parties are not included in the PPDB. However, being electorally successful –that is, having won at least one seat in the national or state parliament in at least one election– is a condition to be part of our crisis party cohort. Another point is that the second round does not include some crisis parties making their breakthrough in national parliaments at coding time (for instance, La France Insoumise or Vox), as Bronet and Borge (2024) warn. Despite this, most parties represented in national parliaments at coding time are included in PPDB Round 2.

In relation to the data of the variables that we consider, we find that a few parties do not have IPD scores. They could not be calculated because there were no available data in PPDB Round 2 to do so. Since three parties do not have AIPD values (all of them crisis) ^5^, for our AIPD analysis, we have considered 26 crisis parties from 13 countries, and all 69 older cohort parties from 13 countries. A total of 95 parties from 13 countries participated in the study. Moreover, the manifesto AIPD dimension (program component) has many missing data ( Brause & Poguntke, 2025b; Von dem Berge & Poguntke, 2017). To control for this fact, there is another AIPD index calculated without the manifesto dimension. Although we will check the differences, the gap between the AIPD index scores and the scores of the AIPD index without the manifesto dimension is very small. We also note that parties that do not have AIPD values do not have PIPD values. In addition, there are three parties that have AIPD values but do not have PIPD values (two crises and one older) ^6^. Hence, for our PIPD analysis, we took into account 24 crisis parties from 13 countries and 68 older cohort parties from 13 countries. A total of 92 parties from 13 countries. With respect to the digitalization index, Bronet and Borge (2024) reported that only three parties (all of them crisis) do not have PPDB data to calculate their digitalization scores ^7^ (all 69 older parties have data).

We consider that the obstacles to the available PPDB data we have just presented are minor and do not represent significant biases for our analysis. In addition, the PPDB is a relevant source of valuable information that can be exploited to compare political parties and thus try to find trends ( Poguntke *et al.*, 2016) as González-Cacheda and Cancela Outeda's (2024) or Bronet and Borge’s (2024) recent works have proved. We believe this project is a great initiative that should continue feeding, updating, and improving.

To answer our second hypothesis (H2) and check the IPD comparison between cohorts, we rely on the first round of the Populism and Political Parties Expert Survey dataset ( Meijers & Zaslove, 2020; Meijers & Zaslove, 2021) which was fielded in 2018 by country experts. We chose the first wave because its data year is in the PPDB round two’s coding time range, and Wave 2 does not provide new information about internal democracy. The POPPA first wave measures the positions and attitudes of 250 parties on key characteristics related to populism, political style, party ideology, and party organization in 28 European countries. In the same way we do with the PPDB, for our analysis we selected political parties from Western European countries and considered as crisis parties those founded from after 2006. The 2018 POPPA does not include any crisis parties from Finland, Sweden, and the United Kingdom, as happens in PPDB Round 1, but there is no crisis party from Norway and Portugal, countries that have crisis parties in the PPDB. Thus, we did not consider older parties' data from these five countries. Instead, the POPPA includes some crisis parties that the PPDB does not, such as La France Insoumise or Compromís. In total, we observed 109 Western European parties from 11 countries, 40 crisis, and 69 older. Consult our shared dataset ( Bronet, 2025) to see the POPPA parties considered, crisis, and older. To select, classify, and compare these parties, we added some of the same variables that we also created in the PPDB: cohort (based on the party foundation year), region of Europe, and left-center-right ideology (based on the party ideology numerical variable of POPPA ^8^). Having done this, we focus on two POPPA variables: intradem ^9^ and populism_cfa_rescaled ^10^. All parties in our sample have populism values and only four parties do not have intradem values ^11^.

Regarding our methodology, we first observe and compare our cohorts through descriptive and bivariate analyses, and then perform multiple regression analyses including all our relevant variables to find the factors that can explain more differences in IPD. We checked the normal distribution of the numerical dependent and independent variables using the Shapiro-Wilk W test and multicollinearity between the independent variables with the VIF measures. We also assessed the degree of alignment between the POPPA manner of measuring internal democracy and the Von dem Berge and Poguntke IPD. Thus, we added the POPPA intradem values to the parties in our PPDB sample, and Spearman’s rank correlation coefficient was calculated between the variables. The first correlation, between the AIPD index and the POPPA internal democracy scale, yielded a coefficient of *ρ* = 0.298 (p = 0.008), while the second, comparing the PIPD index to the same POPPA scale, was slightly higher ( *ρ* = 0.328, p = 0.004). Although the correlations were moderate, both were statistically significant, suggesting a consistent association between the measures. This indicates that despite differences in operationalization, the indicators tend to capture a similar underlying pattern.

## Empirical analysis / results

The first hypothesis argues that the crisis parties are more plebiscitary than older cohorts, especially if the crisis parties are on the left and highly digitalized. In contrast, the older cohort’s parties, especially the old left-wing parties, have higher levels of assembly-based IPD than the crisis parties. Being more plebiscitary than the older cohort’s parties means that the crisis parties have higher PIPD scores than the older parties, the crisis parties have fewer gaps between AIPD and PIPD values (because it could be that the AIPD scores of these parties were lower) than the older parties and/or the crisis parties have PIPD values over AIPD scores. Examining this, we will also examine whether these parties are essentially plebiscitary. This observation also provides information on whether the crisis parties are more democratic than older parties by comparing their AIPD and PIPD values.

**Figure 1.**
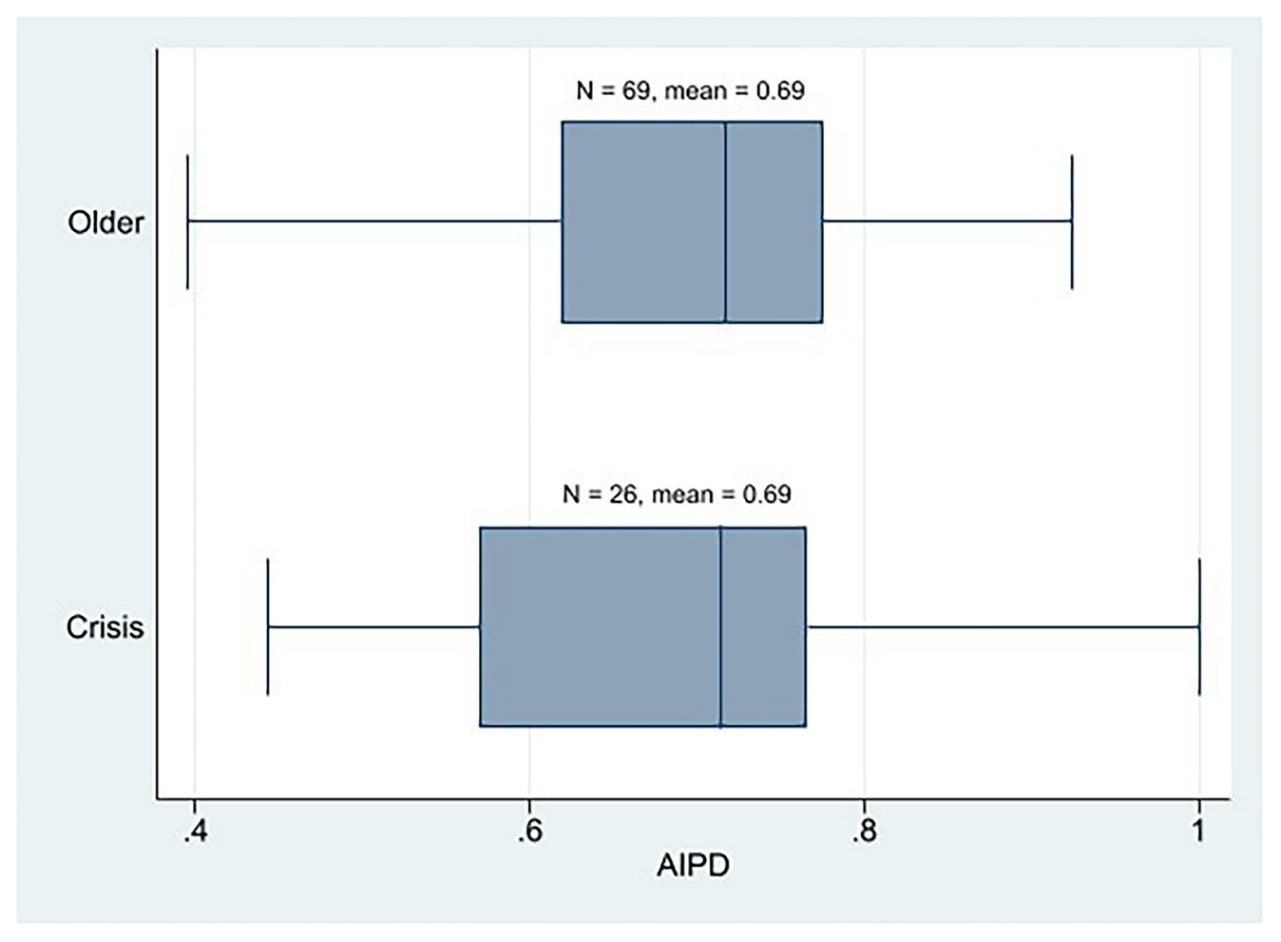
Western European parties AIPD by cohort.

**Figure 2.**
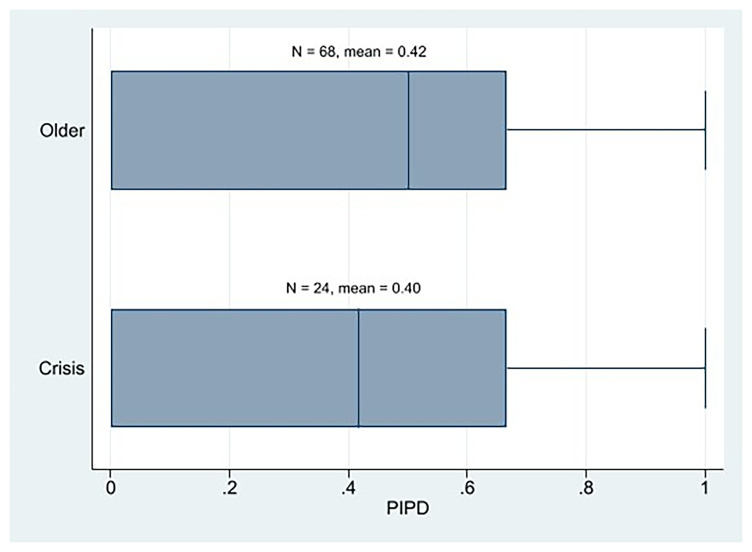
Western European parties PIPD by cohort.

At the descriptive level, we observe that the AIPD mean of the crisis parties is the same as that of the older Western European parties ( Figure 1), and the PIPD mean of the crisis parties is slightly lower than that of the older parties ( Figure 2). In addition, the AIPD mean of the crisis parties was clearly higher than that of its PIPD. Therefore, at first glance, we cannot state that the crisis parties are more plebiscitary than the older parties, nor are the crisis parties essentially plebiscitary. We cannot say that the crisis parties are more democratic than the older parties or the opposite either, because their IPD means (AIPD and PIPD) are similar.

In more detail, only five crisis parties (out of 26) have higher PIPD than AIPD values ^12^. In all these cases, the AIPD scores were also considerably high (from 0.57 to 0.78). Von dem Berge and Poguntke (2017) pointed out that when parties have high levels of AIPD, high levels of PIPD do not necessarily lead to empowering elites. Only three (out of 26) scored 1 in the PIPD index ^13^. Some new parties that literature usually takes as references for observing their internal democracy, such as Movimento 5 Stelle in Italy or Alternativet in Denmark, have higher AIPD scores than PIPD’s. In fact, the only frequently cited case with a higher PIPD than AIPD was Podemos in Spain.

Regarding older parties, 15 out of 69 older parties had higher PIPD than AIPD values and 10 scored 1 in the PIPD index. Both figures represent slightly higher rates than those of the crisis parties. Thus, we can also state that for the crisis parties being essentially plebiscitary would not be the rule; in fact, there is a lower proportion of plebiscitary parties among them than among the older cohort’s parties.

**Figure 3.**
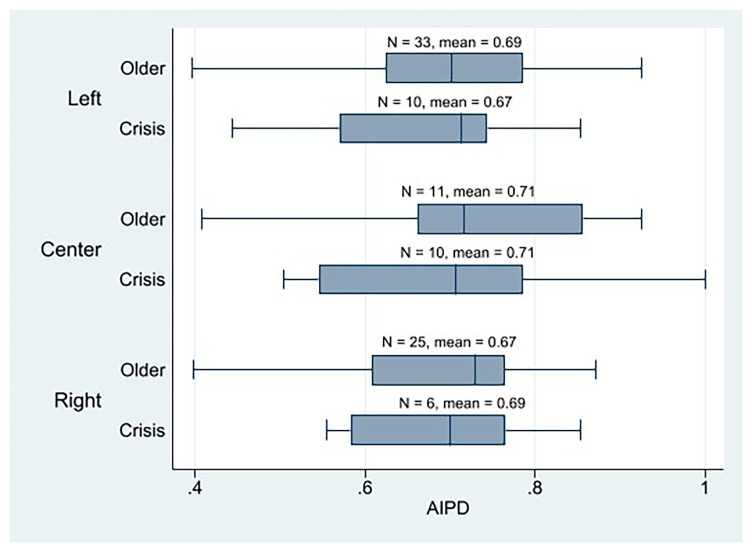
Western European parties AIPD by cohort and ideology.

**Figure 4.**
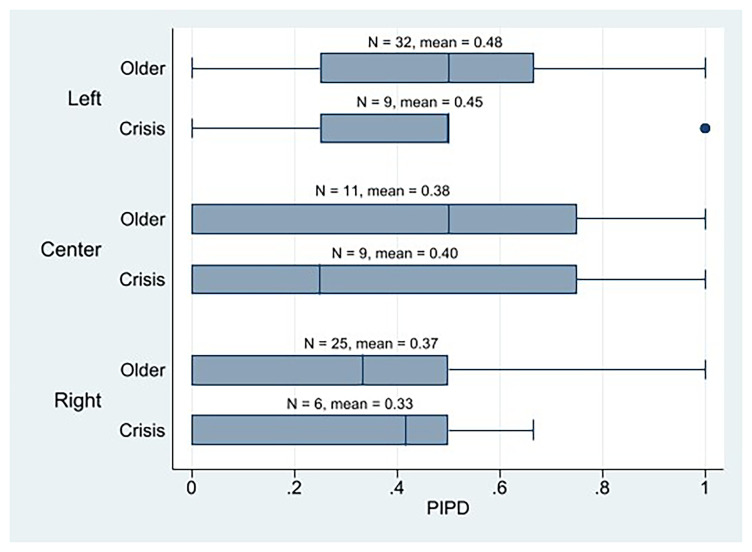
Western European parties PIPD by cohort and ideology.

Concerning ideology, we note that the AIPD mean of all crisis parties’ ideologies are very similar, with the left parties surprisingly scoring the lowest and the center parties the highest ( Figure 3). Instead, left-crisis parties obtain the highest PIPD mean, followed by the center, and ending with the right parties ( Figure 4).

Therefore, by considering only the mean, we might say that the parties from the left wing would be the most plebiscitary parties among the crisis parties because they have the highest PIPD mean, and the difference between PIPD and AIPD is the smallest. Yet, as we can notice in the PIPD boxplot ( Figure 4), this is mainly due to the fact that there are two parties among them that score 1 in PIPD. No other left-crisis party scored above 0.5. Instead, there are four center parties that score over 0.5, in PIPD. We also observe that three out of the five crisis parties that have higher PIPD than AIPD values are center parties, and two are left ones. And two of the three crisis parties that score 1 in the PIPD index are left parties, and one is center. Thus, although their PIPD mean is slightly lower than that of the left-wing parties, the weight center or liberal parties have in the most plebiscitary crisis parties is something remarkable. The AIPD mean of the center crisis parties (and the order’s too) is also notable. Together, these results lead us to believe that center crisis parties show a high score in internal democracy. It is clear that right crisis parties are the least plebiscitary (lowest PIPD scores). In any case, we can see how the crisis parties, in general terms, use the direct vote of all their members as a mechanism to make decisions by complementing their considerably well representative devices.

With all these data, we could not clearly affirm that the crisis parties from the left are more or less democratic than the center or even the right-wing parties. We cannot state that the crisis parties from the left –most of those which claim they bet on boosting their internal democracy to regain legitimacy– are essentially plebiscitary, except in very specific cases ^14^. Having the highest PIPD mean and the smallest gap between PIPD and AIPD among the crisis parties does not have to stand for that they are essentially plebiscitary because the AIPD mean of the crisis parties from the left is prominently higher than PIPD. Moreover, the PIPD mean of the older left-wing parties was even higher than that of the left-wing crisis parties ( Figure 4). The same happens if we compare the PIPD mean of the left crisis parties that are more digitalized ^15^ to the left older cohort parties that are more digitalized ( Table 1). What is true is that among the highest digitalized parties, there is a significant relationship between the left-wing parties and PIPD (p = 0.041), although the highly digitalized crisis parties from the center obtained the highest PIPD mean. However, this cohort did not seem to be an explanatory factor.

**Table 1.** PIPD of the highly digitalized parties by ideology and cohort.

Ideology	N (Crisis)	Mean (Crisis)	SD (Crisis)	N (Older)	Mean (Older)	SD (Older)
Left	7	0.476	0.413	16	0.521	0.321
Centre	6	0.569	0.370	7	0.321	0.345
Right	3	0.333	0.289	12	0.257	0.240

*Note: PIPD stands for Plebiscitary Intra-Party Democracy. Cohort: "Crisis" = Western European parties founded from 2006 onwards; "Older" = Western European parties founded before 2006. Ideology is coded as Left (1), Centre (2), Right (3).*

With regard to the second part of H1 ( *older cohort’s parties, especially the old left-wing parties, have higher levels of assembly-based IPD than the crisis parties.*), we observe that the left older cohort’s parties have a higher AIPD mean than the crisis parties, but there is barely any difference ( Figure 3). It is also noteworthy that center parties (both crisis and older) obtained the highest score in AIPD.

In any case, we must be cautious of these descriptive results because the number of observations for all segmented groups is small. 

To determine whether the observed variables can explain the IPD level and form of political parties in Western Europe, we carried out two multiple regression analyses. In the first one, the dependent variable is the AIPD index ( Table 2), and in the second, the PIPD index ( Table 3). The explanatory variables are cohort (being a crisis party or an older party), ideology, and the level of digitalization (numerical -0 to 1-). The metrical variables follow a normal distribution ^16^ and there is no multicollinearity ^17^. Due to the limited sample size, especially for crisis parties (n = 26 for AIPD and n = 24 for PIPD), we resort to bootstrapping to improve the robustness of the estimates. It is a resampling method that relaxes distributional assumptions and is well-suited for small samples. Below is a summary of the regression analysis results.

**Table 2.** Linear regression predicting AIPD. Bootstrapped standard errors (1,000 replications).

Variable	Coefficient	Std. Error	z	p-value	95% CI
Cohort	-0.023	0.030	-0.76	0.448	[-0.082, 0.036]
Digitalization	0.291	0.087	3.33	0.001 ***	[0.120, 0.462]
Ideology	-0.0004	0.014	-0.03	0.978	[-0.028, 0.027]
Constant	0.563	0.051	11.08	0.000 ***	[0.464, 0.663]

**Model statistics:** N = 93       R² = 0.133       Adj. R² = 0.104       Root MSE = 0.124       Prob > χ² = 0.0076 **Notes:**     •   Dependent variable: *AIPD* (Assembly-based Intra-Party Democracy index).     •   Standard errors are based on 1,000 bootstrap replications.     •   ***p < 0.01.

**Table 3.** Linear regression predicting internal PIPD. Bootstrapped standard errors (1,000 replications).

Variable	Coefficient	Std. Error	z	p-value	95% CI
Cohort	-0.045	0.077	-0.59	0.556	[-0.196, 0.106]
Digitalization	0.335	0.172	1.94	0.052 *	[-0.003, 0.672]
Ideology	-0.058	0.039	-1.48	0.138	[-0.135, 0.019]
Constant	0.386	0.110	3.50	0.000 ***	[0.170, 0.602]

**Model statistics:** N = 91      R² = 0.052      Adj. R² = 0.019      Root MSE = 0.337      Prob > χ² = 0.119 **Notes:**     •   Dependent variable: *PIPD* (Plebiscitary Intra-Party Democracy index).     •   Standard errors are based on 1,000 bootstrap replications.     •   *p < 0.1, ***p < 0.01.

Digitalization emerged as a significant coefficient for both the AIPD and PIPD indices. The other variables included in our model (being a crisis party or an older party and ideology) do not influence the degree and form of intra-party democracy. Therefore, the level of digitalization is linked to the IPD levels. The more digitalized a Western European party is, regardless of whether it is crisis or older, left-wing or right-wing, the more internally democratic it is.

Our second hypothesis states that populism is a factor that nuances the previous hypotheses in the sense that makes left-wing crisis parties that are populist to be less democratic than older left-wing parties, though are more democratic than the populist right ones.

**Figure 5.**
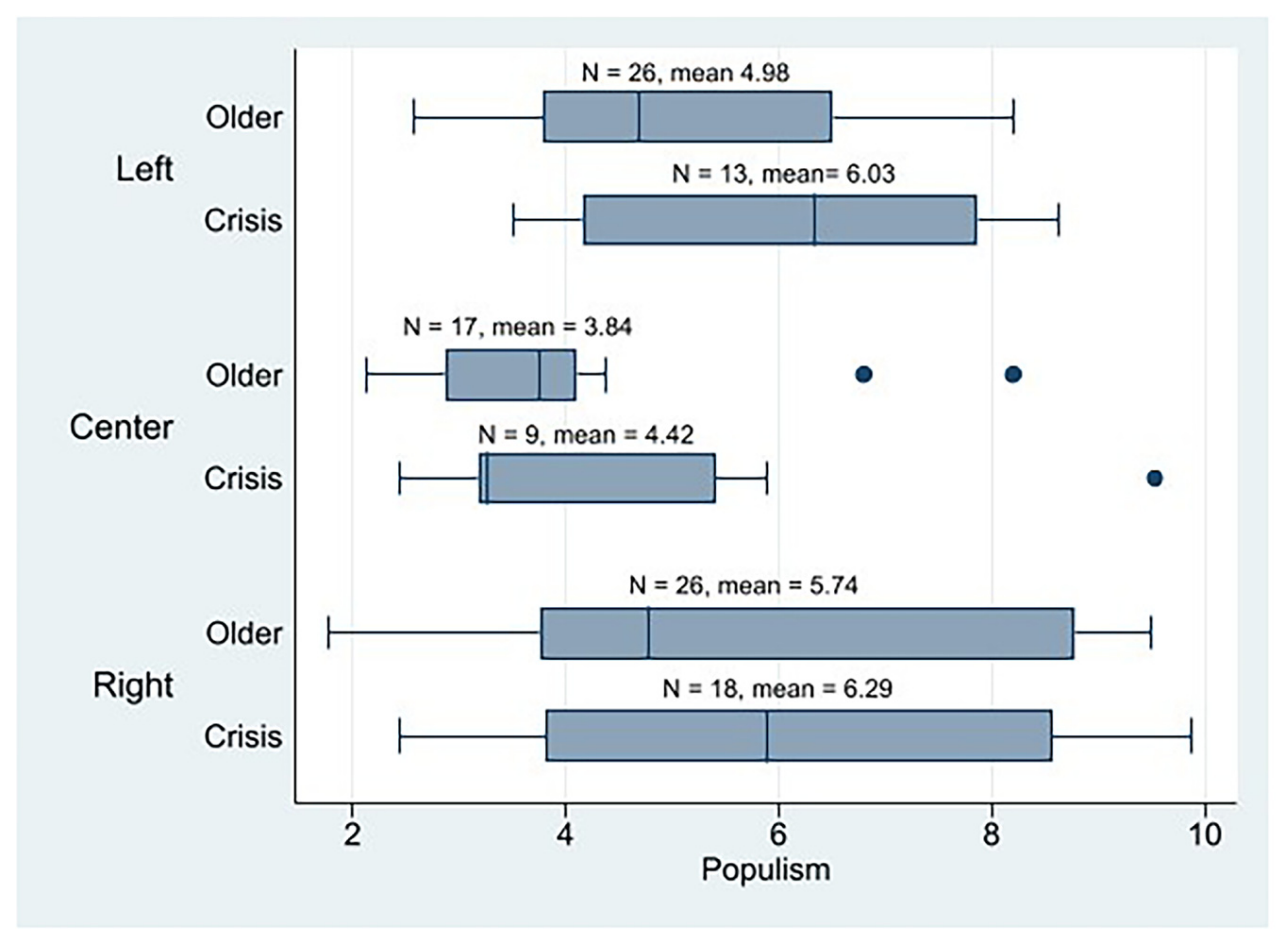
Western European parties populism by cohort and ideology.

At the descriptive level, initially, we observe that the crisis parties in general are more populist (n = 40, mean = 5.79) than the older cohorts (n = 69, mean = 4.99). In Figure 5, we can observe that the crisis parties of all three ideologies are more populist than their older counterparts by considering their means. Moreover, 23 out of 40 crisis parties scored 5 or more on the populism scale (58%), which could be referred to as populists, while 24 out of 69 older parties do (35%). This means that the weight of populist parties in the crisis parties is much bigger than in the older parties. Therefore, the crisis parties would be more populist than the older cohort’s parties, as Katz (2021) points out when discussing the increase in anti-party-system parties. As shown in Figure 5, ideology appears to be a relevant factor that explains populism as well (being the center parties the least populist).

**Figure 6.**
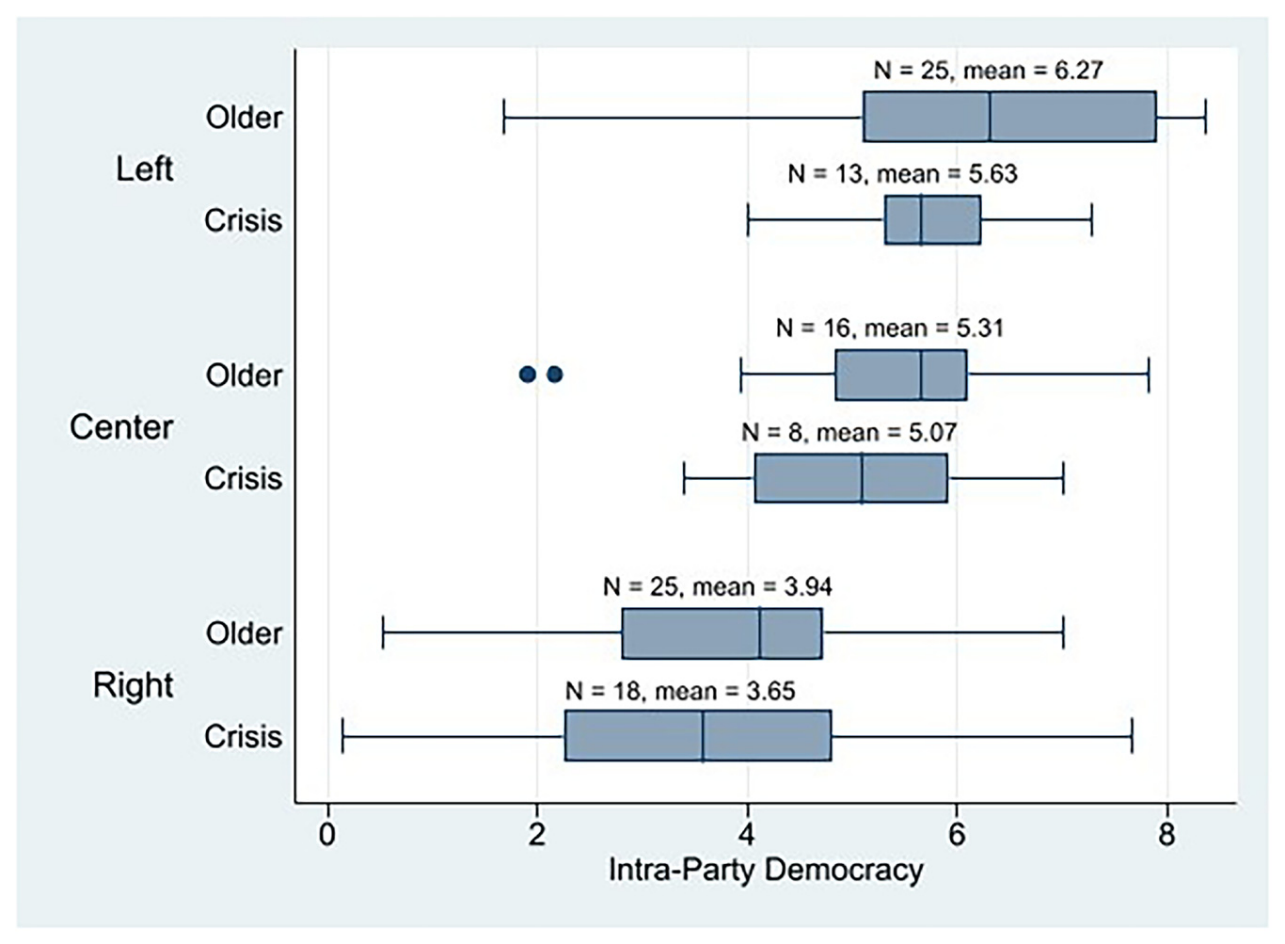
Western European parties IPD by cohort and ideology.

Thus, if intra-party democracy is negatively correlated with populism ( Böhmelt *et al.*, 2022) and the crisis parties are more populist than the older cohort parties, the crisis parties should be less internally democratic than the older parties. That is what their intra-party means show us, with the crisis parties’ being slightly lower (n = 39, mean = 4.60) than the older parties’ (n = 66, mean = 5.15). Figure 6 shows how ideology combines with cohorts (affected by populism) to shape internal democracy. The left-wing old parties would be the most internally democratic, followed by the left-wing crisis parties, and the right crisis parties closing. The IPD mean of the crisis parties across all three ideologies was lower than that of their older counterparts.

If we consider only the populist ^18^ crisis parties, we find a significant relationship between ideology and intra-party democracy IPD (p = 0.000), with the left-crisis parties (n = 8, mean = 5.49) being more democratic than the center (n = 3, mean = 4.64) and right-crisis populist parties (n = 12, mean = 2.80). As with the descriptive analysis of the PPDB dataset, these results must be considered with caution because, in some cases, the size of the crisis parties’ subgroups is very small.

In the same way we have done for the H1, we carried out a multiple regression analysis with our POPPA sample ( Table 3) to study how our independent variables may influence the internal democracy. The dependent variable is the internal democracy index (numerical -0 to 10-), and the explanatory variables are cohort (dummy in which the reference category is older party), ideology (dummy in which the reference category is center), and level of populism (numerical -0 to 10-). The internal democracy index followed a normal distribution ^19^, but the populism level did not, ^20^ and there was no multicollinearity ^21^. Although the distribution of the populism variable is not strictly normal, normality is not required for independent variables in linear regression. What matters is the distribution of the residuals, which is handled in this case using bootstrapped standard errors. This approach avoids reliance on parametric assumptions including normality. In addition, the populism variable is rescaled and used in its form by Böhmelt *et al.* (2022). Table 4 provides a summary of the regression analysis.

**Table 4.** Linear regression predicting internal democracy. Bootstrapped standard errors (1,000 replications).

Variable	Coefficient	Std. Error	z	p-value	95% CI
Crisis parties	-0.032	0.258	-0.13	0.900	[-0.537, 0.473]
Left (vs. Centre) ideology	1.445	0.319	4.53	0.000 ***	[0.820, 2.071]
Right (vs. Centre) ideology	-0.425	0.318	-1.34	0.181	[-1.048, 0.198]
Populism	-0.497	0.060	-8.29	0.000 ***	[-0.614, -0.379]
Constant	7.251	0.332	21.82	0.000 ***	[6.600, 7.903]

**Model statistics:** N = 105       R² = 0.591       Adj. R² = 0.574       Root MSE = 1.239       Prob > χ² = 0.000 **Notes:**     •   Dependent variable: *intradem* (internal democracy index).     •   Reference category for ideology: Centre.     •   ***p < 0.01.     •   Standard errors based on 1,000 bootstrap replications.

With our sample of only Western European parties, one can observe what Böhmelt *et al.* (2022) already found for all parties included in the POPPA, in the sense that ideology and populism affect internal democracy. There is a positive correlation between being a left-wing party and internal democracy, as well as a negative correlation between populism and internal democracy. Thus, we can confirm what is observed with the descriptive statistics, populism, and ideology (being a left party) are significant variables that may explain intra-party democracy with very good capacity (high R²) in our model. Instead, the cohort does not seem to directly influence IPD. Nevertheless, the cohort affects intra-party democracy to the extent that crisis parties are more populist than older parties, so they are less internally democratic. When we only examined the crisis parties, both correlations (ideology and populism) persisted ( Table 5).

**Table 5.** Linear regression predicting internal democracy among crisis parties. Bootstrapped standard errors (1,000 replications).

Variable	Coefficient	Std. Error	z	p-value	95% CI
Left (vs. Centre) ideology	1.169	0.589	1.99	0.047 **	[0.015, 2.324]
Right (vs. Centre) ideology	-0.700	0.584	-1.20	0.231	[-1.845, 0.446]
Populism	-0.397	0.108	-3.67	0.000 ***	[-0.610, -0.185]
Constant	6.857	0.700	9.79	0.000 ***	[5.485, 8.230]

**Model statistics:** N = 39       R² = 0.512       Adj. R² = 0.470       Root MSE = 1.295       Prob > χ² = 0.000 **Notes:**     •   Dependent variable: *intradem* (internal democracy index).     •   Reference category for ideology: Centre.     •   **p < 0.05, ***p < 0.01.     •   Standard errors based on 1,000 bootstrap replications.

Overall, we can confirm H2, that is, by being more populist, left-wing crisis parties are less democratic than older left-wing parties, although they are more democratic than the crisis right ones.

## Discussion and conclusions

Our empirical analysis, based on a considerable number of political parties ^22^, sheds more light on the debate around how the internal democracy of Western European young parties is by contrasting what the most relevant literature has found so far, mainly through case studies.

Bronet and Borge (2024) developed a generational approach to delimit the phenomenon of the boom of electorally successful new political parties in Western Europe over the past two decades, which allowed us to establish our cohorts (crisis parties and older parties). Von dem Berge and Poguntke (2017) offer a model to measure the internal democracy of political parties that distinguishes between assembly-based and plebiscitary intra-party democracy. Finally, the PPDB and the POPPA provide us with data to be able to analyze the IPD of the crisis parties, compare it among them, and to the older parties. By operationalizing our datasets within this framework, we check the two hypotheses we formulate based on the literature.

First, regarding our first hypothesis, we may reject it as a result of our descriptive analysis, which shows that the crisis parties are neither more plebiscitary than the older Western European parties nor are they essentially plebiscitary. For the crisis parties, being essentially plebiscitary is not the rule. In fact, there are fewer plebiscitary parties among them than among older Western European parties. We neither may say that the crisis parties from the left, most of which claim they bet on boosting their internal democracy to regain legitimacy, are essentially plebiscitary except in very specific cases (the AIPD mean of the crisis parties from the left is prominently higher than PIPD). The older parties from the left would be the most plebiscitary (they have the highest PIPD mean, and the difference between PIPD and AIPD is the smallest among all parties). What is true is that left-wing parties seem to be the most plebiscitary parties among the crisis parties, although the weight center parties have in the most plebiscitary crisis parties is remarkable. Not even the left-wing crisis parties that are highly digitalized are the most plebiscitary parties; they are the crisis parties from the center, followed by the older parties from the left. Lastly, regarding the latter assertion of H1, the older cohort’s parties do not have higher levels of assembly-based IPD than crisis parties. Not even the old left-wing parties have a high AIPD; center parties (both crisis and older) score the highest. Notwithstanding, the results of these descriptive IPD results should be interpreted cautiously because, in some cases, the size of the crisis party subgroup is very small.

The information we observe by comparisons among parties from our sample contrasts with previous literature, mostly based on case studies, which led us to formulate our first hypothesis, in the sense that we might expect that the internal democracy of the crisis parties from the left that are highly digitalized would adopt a plebiscitary form ( Barberà & Rodríguez-Teruel, 2020; Biancalana, 2016; Gerbaudo, 2019; Invernizzi-Accetti & Wolkenstein, 2017; Rodríguez-Teruel & Barberà, 2018). In fact, the only oft-mentioned party that has a higher PIPD than AIPD is Podemos (Spain). Nonetheless, as we note before, we must consider that case studies may provide detailed factors that PPDB, essentially based on party statutes (normative dimension), does not include.

By applying our regression model to our PPDB data, we found that digitalization (in terms of more web affordances) has an impact on IPD (both AIPD and PIPD), while being a crisis or an older party and ideology do not seem to be significant in increasing internal democracy. Thus, the more digitalized a Western European party is, the more internally democratic it is. Digitalization is seen as a good condition or possibility of improving internal democracy.

Second, the descriptive analysis of our POPPA sample initially shows that the crisis parties are more populist than the older cohort parties from Western Europe, as Katz (2021) points out when discussing the increase in anti-party-system parties. However, the crisis parties of all three ideologies (left, center, and right) are more populist than their older counterparts are. In addition, there is a greater proportion of what we can call populist parties among the crisis than among the older parties. Thus, if populism is negatively correlated with intra-party democracy, crisis parties would be less democratic than the older cohorts. And that is it. In general, crisis parties are slightly less internally democratic than older parties. We also observed that ideology, combined with cohorts (affected by populism), plays an important role in shaping internal democracy. Left-wing parties are the most internally democratic among the crisis parties (and the second ones, including the older parties) but are less internally democratic than the left-wing older parties. If we only consider populist crisis parties, ideology shows a strong relationship with the IPD. The left-wing populist crisis parties would be more democratic than the center and right crisis populist parties. Therefore, we can confirm H2, that is, by being more populist, left-wing crisis parties are less democratic than older left-wing parties, although they are more democratic than crisis right parties. We want to remind us that these results should be considered with caution because when we segment our sample, some subgroups have few observations.

With the regression analysis of our POPPA dataset, we can confirm that populism and ideology (being a left party) remain sounded explanatory variables for intra-party democracy when we focus only on Western European parties, as Böhmelt *et al.* (2022) already found for all the parties included in the POPPA. Instead, the cohort does not seem to directly influence IPD. Cohort affects internal democracy to the extent that crisis parties are generally more populist than older parties, so they are less internally democratic.

In summary, with the theoretical framework and datasets we have used, we cannot state that being a crisis party, even a highly digitalized one from the left, results in a more plebiscitary internal democracy. The point is that the crisis parties are more populist than the older cohort parties, a fact that pushes down their internal democracy.

Von dem Berge and Poguntke’s IPD model has proved useful for our purposes and appears sound, relating our findings to previous research on this topic. It is also true that this model, which is based mainly on party statutes (normative dimension), does not consider the detailed factors of intra-party democracy, information that case studies can provide. In this sense, our empirical work aims to complement the qualitative research. POPPA has proven to be a good dataset to verify some results obtained from the PPDB and to introduce populism as an IPD explanatory variable. POPPA Wave 1 was coded in the same time range as PPDB Round 1 and includes more crisis parties than PPDB, allowing us to increase the sample of the units we aim to study. Although the POPPA internal democracy measurement does not distinguish between AIPD and PIPD, both theoretically and operationally appears to be well-aligned with Von dem Berge and Poguntke’s IPD model. At this point, we would like to emphasize the importance of continuing to feed, update, and improve cross-national projects such as PPDB and POPPA in order to collect more and better data for quantitative research that helps us compare political parties.

Finally, we see an opportunity to continue and complement our empirical analysis. We consider it interesting to know how the internal democracy of the crisis parties is evolving to determine whether these parties, as a result of the institutionalization process, are becoming less democratic or more plebiscitary. In addition, IPD variations over time may be related to electoral support. Crisis parties may have lost seats in national parliaments because they were becoming institutionalized and reducing their IPD. In this sense, the upcoming third round of the PPDB may allow us to answer these questions. This work could also be enriched with surveys, focus groups, and interviews with members and leaders of some crisis parties to obtain their views and perceptions of the evolution of their internal democracy over time.

## Ethics and consent

Ethical approval and consent were not required.

## Data Availability

All data and materials are available in the Open Science Framework. Root data **DOI:** https://doi.org/doi:10.7910/DVN/0JVUM8 ( Scarrow *et al.*, 2022b). Political Party Database Round 2 v4 (First Public Version). Harvard Dataverse. Data are available under the terms of the Creative Commons Zero “No rights reserved” data waiver (CC0 1.0 Public domain dedication). **DOI:** https://doi.org/doi:10.7910/DVN/GKFIV3 ( Brause & Poguntke, 2025a). Intra-Party Democracy Indices (V1) Based on PPDD Round 2. Harvard Dataverse. Data are available under the terms of the Creative Commons Zero “No rights reserved” data waiver (CC0 1.0 Public domain dedication). **DOI:** https://doi.org/doi:10.7910/DVN/RMQREQ ( Zaslove *et al.*, 2024). Populism and Political Parties Expert Survey 2023 (POPPA). Harvard Dataverse. Data are available under the terms of the Creative Commons Zero “No rights reserved” data waiver (CC0 1.0 Public domain dedication). New data Repository: CORA.Research Data Repository. https://doi.org/10.34810/data2523 ( Bronet, 2025). This dataset contains the following files: PPDB_R2_Crisis Parties. (PPDB Round 2 of the political parties from the Western European countries that include Crisis Parties with these new variables added at the end: year of party foundation, cohort -crisis/older party-, region of Europe, left-center-right ideology, digitalization index, aipd, pipd, oipd, aipd_wm, and POPPAintradem). Original data are in STATA format. Poppa_W1_Crisis Parties. (POPPA Wave 1 of the political parties from the Western European countries that include Crisis Parties with these new variables added at the end: cohort -crisis/older party-, region of Europe, left-center-right ideology). Original data are in STATA format. Data are available under the terms of the Creative Commons Zero "No rights reserved" data waiver (CC0 1.0 Public domain dedication).

## Footnotes

^1^ The only consultation that has taken place when Podemos has been in government was related to the negotiations to join the SUMAR coalition for the 2023 general elections. The consultation was called from one day to the next, lasted less than 24 hours (from 1:30 p.m. on June 8 to 10 a.m. on June 9), and the question sought to empower its leaders (“Do you accept that the Coordination Council of Podemos, following the criteria of unity that decided the State Citizen Council, may negotiate with SUMAR and, if necessary, agree to an electoral alliance between Podemos and Sumar?”). The support received was 92.92%.

^2^ That is still open but not linked to the Podemos’ website and which has become a forum for complaints and protest against the leaders and the way Podemos functions.

^3^ One to strengthen its autonomy within the coalition SUMAR in November 2023 (86% of participants supported the leaders position) and another one, in October 2024, to condition its support for the General State Budget on some demands (89.8% of participants supported the leaders position).

^4^ Austria, Belgium, Ireland, Spain, Portugal, United Kingdom, Switzerland, France, Denmark, Finland, Germany, Italy, Netherlands, Norway, Greece, Sweden.

^5^ Independents4Change and Solidarity-People Before Profit, both left-wing parties from Ireland, and Party for Freedom, a right party from the Netherlands.

^6^ Liberal Alliance (center party from Denmark), DENK (left party from the Netherlands) and Ecologist Party "The Greens" (left party from Portugal).

^7^ Parti Populaire (right party from Belgium), the Liberal Alliance (center party from Denmark) and Independents4Change (left party from Ireland).

^8^ >= 4 left, >4 & <6 center, and >= 6 right on the “lroverall” variable scale.

^9^ POPPA item: “Some political parties practice more intra-party democracy than others (i.e., party members’ play a role in decision-making, there is room for internal debate, decision-making is inclusive of various factions and organizational layers within the party)”. Experts rate parties on a scale from 0 to 10, with 10 standing for a very high level of intra-party democracy (0 stands for no intra-party democracy at all).

^10^ It captures the degree of populism for each political party in the data as taken from POPPA. This variable is a weighted arithmetic mean of its five subcomponents: “manichean”, "indivisible", “generalwill”, “peoplecentrism”, and “antielitism”. It ranges between 0 (a party is not at all populist) and 10 (a party is very populist).

^11^ DENK (Netherlands), as a crisis party, and Canary Coalition (Spain), Federal Democratic Union of Switzerland (Switzerland) and Swiss Party of Labour (Switzerland), as old parties. Two left-wing parties, one center and one right.

^12^ NEOS (Austria), Social Democrats (Ireland), PDECAT (Spain), Podemos (Spain) and Ciudadanos (Spain).

^13^ NEOS (Austria), Social Democrats (Ireland), Podemos (Spain).

^14^ Only 2 out of 10 left crisis parties, Podemos (Spain) and Social Democrats (Ireland) have higher PIPD values than AIPD. Both score 1 (the maximum) in the PIPD index.

^15^ 51 parties that score 0.5 or more in the digitalization index variable, i.e., that have values manifestly over the mean (0.4).

^16^ AIPD Shapiro–Wilk W = 0.98, p = 0.14; PIPD Shapiro–Wilk W = 0.99, p = 0.84; digitalization Shapiro–Wilk W = 0.99, p = 0.99.

^17^ AIPD and PIPD regression VIF of independent variables < 1.5.

^18^ Parties that score 5 or more in the “populism_cfa_rescaled” variable.

^19^ Intra-party democracy Shapiro–Wilk W = 0.98, p = 0.22.

^20^ Populism Shapiro–Wilk W = 0.91, p = 0.00.

^21^ VIF of all independent variables < 2.

^22^ 98 from the PPDB and 109 from the POPPA.
