# The internal democracy of the crisis parties in Western Europe: a quantitative analysis of the role of digitalization, ideology and populism.

**DOI:** 10.12688/openreseurope.21115.2

**Published:** 2025-12-16

**Authors:** Jorge Bronet, Rosa Borge

**Affiliations:** 1 Communication Networks & Social Change research group (CNSC),UOC-TRANSIC Centre, Open University of Catalonia, Barcelona, Catalonia, 08018, Spain

**Keywords:** new political parties, intra-party democracy, participation, digitalization, populism, Western Europe.

## Abstract

**Background:**

Previous studies on the internal democracy of new digital parties in Western Europe suggest a plebiscitary tendency, but most focus on a limited number of cases. This paper aims to empirically analyze the intra-party democracy of electorally successful new parties in Western Europe and identify the main factors that may influence it.

**Methods:**

Drawing on data from the second round of the Political Parties Database (PPDB) and the first wave of the Populism and Political Parties Expert Survey (POPPA), this study covers more than 100 parties across 13 countries. Adopting a generational approach, we define a cohort of “crisis parties”—founded between the economic crisis and the pandemic—and examine their internal democracy in comparison to older parties, using Von dem Berge and Poguntke’s IPD model and Böhmelt *et al.*,’s (2022) framework, with ideology, digitalization, and populism treated as explanatory variables.

**Results:**

Our findings show that being a crisis party—even a highly digitalized one on the left—does not entail more plebiscitary forms of intra-party democracy.

**Conclusions:**

Digitalization emerges as the most consistent predictor shaping intra-party democracy, while the cohort effect matters only insofar as crisis parties are more populist than older parties, which ultimately reduces their internal democracy.

## Introduction

Over the last two decades, many new parties have arisen in Western Europe. The emergence of new parties is not a novelty, but their number and electoral success, especially in some countries, are particularly relevant during this period ( Chiaramonte & Emanuele, 2022). Apparently, some of these parties, at least in their first stage, implemented measures to enhance their internal democracy as a means of re-engaging disaffected citizens, often leveraging digital technologies. Nevertheless, many authors point out that these parties are subject to electoral competition and become institutionalized, a fact that makes them take steps backwards in terms of internal democracy (for instance, Meloni & Lupato, 2023). Some scholars also claim that their participation model is essentially plebiscitary (the what, how, and when of what is discussed or asked remain in the hands of an elite), which triggers the effect of reinforcing the power of leaders and failing to empower members and citizens ( Barberà & Rodríguez-Teruel, 2020; Biancalana, 2016; Gerbaudo, 2019; Invernizzi-Accetti & Wolkenstein, 2017; Rodríguez-Teruel & Barberà, 2018). However, the analysis of Von dem Berge and Poguntke (2017) shows that plebiscitary intra-party democracy (PIPD) appears to be a complement for parties with high assembly-based intra-party democracy (AIPD) and parties with high levels of PIPD and low levels of AIPD are extremely unusual.

A common observation in the current scientific literature is the prevalence of descriptive case studies ( Correa *et al.*, 2021). Indeed, most studies on the Intra-Party Democracy (IPD) of new European parties focus on a single case or a limited number of parties ( Biancalana, 2016; Borge & Santamarina Sáez, 2016; Deseriis, 2020; Gad, 2020; Gerbaudo, 2019; Gherghina & Stoiciu, 2020; Rodríguez-Teruel & Barberà, 2018; Vodová & Voda, 2020). Data availability concerning these emerging political parties, specifically drawn from the Political Parties Database round 2 ( Poguntke *et al.*, 2022; Scarrow *et al.*, 2022b) and the Populism and Political Parties Expert Survey dataset (POPPA database, Meijers & Zaslove, 2020; Meijers & Zaslove, 2021), enables the utilization of a quantitative, large-N research design. This approach is vital for systematic hypothesis testing, allowing for the rigorous verification of patterns established in previous qualitative scholarship. Furthermore, it provides the capacity to yield novel, comparative insights within the geographically and politically constrained framework of Western Europe. This paper seeks to contribute to the ongoing academic discourse concerning the nature and determinants of IPD within the new political parties of Western Europe by examining the interplay between digitalization, ideology, and populism. A central aim of this investigation is to empirically assess two key research questions: Are these newer political formations demonstrably more democratic than their established counterparts? and In what ways, and why, does their IPD diverge from traditional party structures? We present new empirical evidence designed to provide a more comprehensive and nuanced response to these questions.

Our conceptualization of IPD places a primary emphasis on inclusiveness, defined as the breadth of the circle of party decision-makers ( Scarrow, 2005). As established by Von dem Berge and Poguntke (2017), inclusiveness constitutes the most salient dimension for evaluating the democratic quality of intra-party decision-making processes, as it inherently incorporates the critical elements of participation, centralization, and accountability. This particular focus on inclusiveness is not merely a methodological choice; it directly addresses the substantive question of whether these new parties genuinely represent a democratic innovation by expanding the opportunities for a wider range of members to participate in decision-making and by modifying the mechanisms through which those decisions are executed. Intra-party democracy is recognized as a contested and multidimensional construct, extending beyond mere deliberation to encompass decision rules, power distribution, and internal accountability. Consequently, this analysis is grounded in a clear delineation of the core elements constituting IPD within the scope of this study. Acknowledging that decision-making represents the most critical dimension of IPD ( Von dem Berge & Poguntke, 2017), the framework primarily (though not exclusively) focuses on the locus of who has the final say across three key areas: organizational structure (concerning the party congress, executive body, and party leader), personnel (including leadership and candidate selection), and program (such as manifestos and policy ballots).

Drawing on the generational approach proposed by Bronet and Borge (2024) to define our case selection (cohort), and the IPD model developed by Von dem Berge and Poguntke (2017) to operationalize the observation of internal democracy, this study analyzes a total of 98 Western European political parties from 13 countries. The sample comprises 29 new and 69 established parties sourced from the Political Parties Database (PPDB). To validate and supplement the findings, a secondary analysis was performed on 109 Western European parties from 11 countries (40 new and 69 traditional parties), using data operationalized from the Populism and Political Parties Expert Survey dataset ( Meijers & Zaslove, 2020; Meijers & Zaslove, 2021).

The article is structured as follows. In the next section, we review and discuss the most relevant literature on the internal democracy of the new Western European parties. Section three establishes the theoretical framework utilized to delineate the phenomenon of new Western European parties (case selection) and operationalize the measurement of intra-party democracy. In section four, we formulate our hypotheses regarding the internal democracy of these parties, considering different explanatory variables, such as cohort, ideology, digitalization, and populism. Section five details the datasets and methodology employed. In section six, we provide the results of our analysis by empirically verifying our hypotheses. Finally, in the last section, we summarize our main findings and suggest a new complementary research line.

## Internal democracy in new parties of Western Europe: the existing evidence

Scholarly inquiry consistently identifies ideology, digitalization, and populism as highly recurrent determinants of intra-party democracy. Furthermore, the novelty of the political party itself—specifically, its recent formation—constitutes another variable that has attracted considerable attention. Consequently, this section provides a brief examination of the scholarly analysis pertaining to the observed effects of these determinants on the IPD of young Western European political parties, which constitutes the empirical focus of this study.

### Ideology, digitalization, and the promise of IPD

Prior research into the IPD of recent political formations has predominantly focused on left-leaning parties (or at least those exhibiting distance from the political right) and those simultaneously characterized by high digitalization (e.g., Podemos, Movimento 5 Stelle, Pirate parties). This focus is premised on the widely accepted assumption that left-wing parties generally exhibit a greater propensity for internal democracy ( Pettitt, 2012; Poguntke *et al.*, 2016; Sandri *et al.*, 2025). Furthermore, the integration of digital technology within these parties is observed to facilitate increased membership participation ( Blasco, 2021; Correa *et al.*, 2021; Raniolo *et al.*, 2021; Vaccari, 2013), and to enable broader plebiscitary voting mechanisms ( Scarrow *et al.*, 2022a).

In addition, several new Western European parties, particularly those on the left, explicitly sought to expand participation and decentralize power by leveraging digital tools. Their emergence (e.g., Podemos, Movimento 5 Stelle, Alternativet) generated significant enthusiasm, as they were often founded on the premise of being truly democratic in contrast to the criticized, hierarchical structure of established parties.

### The institutionalization dilemma: Centralization vs. Participation

Despite their initial democratic mandates, a significant body of literature suggests a decline in the initial participation rate and a subsequent retreat from decentralized power as these new parties institutionalize ( Bennett *et al.*, 2018; Gad, 2020; Invernizzi-Accetti & Wolkenstein, 2017; Lioy *et al.*, 2019; Rodríguez-Teruel & Barberà, 2018). This regression is frequently attributed to the tension between two opposing forces: the original intent to increase internal participation and deliberation and the need to centralize power to effectively compete at the polls. This observation aligns with Kitschelt’s (2006) view of movement parties as transitional phenomena on their way to becoming centralized political entities.

Central to this dilemma is the widely argued principle that electoral competition rewards centralized, hierarchical structures. Strom (1990) famously posited that the decentralization of political decision-making is detrimental to vote maximization. Several scholars warn that greater efforts toward participation may negatively impact electoral outcomes (e.g., Fitzpatrick, 2018), while leader-centered parties pursuing a vote-seeking strategy tend to perform better. Consequently, the demands of electoral success may pressure parties toward oligarchic structures ( Löfgren & Smith, 2003). Struggling to reconcile internal democratic practices with cartelization strategies—employed to fortify leadership and enhance systemic competitiveness—these emergent parties exhibit an organizational form that is suboptimal for effective political competition (lacking a vertical structure) and misaligned with the demands of their electorate and activists ( Bennett *et al.*, 2018).

The challenge facing these parties, especially following declines in electoral support (for instance, Podemos, Syriza, Movimento 5 Stelle), is how to institutionalize without undermining the IPD that forms the basis of their existence and electoral appeal ( Lioy *et al.*, 2019). Rapid growth and the shift toward maximizing public office (e.g., entering coalition governments) may lead to a modification of their initial strategy ( Harmel & Janda, 1994).

Case studies, such as the analysis of Podemos (Spain) by Meloni and Lupato (2023), compellingly exemplify the tension between interactivity and the need for control, detailing regressions in the use of digital innovations for IPD that were driven by party decisions, not technical limitations.

### Top-down control

In a broader sense, relevant literature has highlighted that the new, predominantly left-leaning parties have demonstrated a limited transfer of power to their constituents ( Lioy *et al.*, 2019; Rodríguez-Teruel & Barberà, 2018). Their participation models are often characterized as essentially top-down ( Gerbaudo, 2019), plebiscitary, aggregative or reactive ( Guglielmo, 2021), where the ability of members to influence the political agenda is low. This structure, where the agenda is controlled by an elite, reinforces the power of leaders ( Barberà & Rodríguez-Teruel, 2020; Biancalana, 2016; Gerbaudo, 2019; Invernizzi-Accetti & Wolkenstein, 2017; Rodríguez-Teruel & Barberà, 2018).

Nevertheless, Von dem Berge and Poguntke (2017) observed a tendency for high levels of plebiscitary IPD (PIPD) to complement high levels of assembly-based IPD (AIPD), with parties exhibiting low AIPD and high PIPD scores being exceptionally rare. Critically, this finding is based on data that excluded nascent political parties (since none were incorporated into the first round PPDB data collection utilized by the authors, despite some having been formed). Consequently, it remains unknown whether contemporary young parties adhere to this pattern or may exhibit an elevated prevalence of PIPD decoupled from a commensurate level of AIPD.

### Populism, and the right wing

The nature of populist parties—a category encompassing many new political formations in Western Europe—logically suggests a prominent reliance on plebiscitary or direct forms of intra-party democracy ( Poguntke *et al.*, 2016). Yet, contrary to the expectation that the ideational form of populist parties would lead to greater internal democracy, Böhmelt *et al.* (2022) found a significant negative relationship between populism and IPD. They observed that populist European parties are generally “undemocratic.” The more populist a party is, the less internally democratic it is, and the more leader-centric it is, although left-wing populist parties exhibited comparatively higher IPD than their right-wing counterparts.

Fewer studies have focused on the internal organizational dynamics of new right-wing parties. While general trends suggest left-wing parties are more internally democratic, the quantitative analysis conducted by Poguntke *et al.*’s (2016) reveals a more nuanced empirical reality. Specifically, their findings indicate that far-right parties possess levels of assembly-based IPD that are comparable to those observed in left-socialist parties, and may even demonstrate a higher prevalence of plebiscitary forms of IPD. This is supported by case studies ( Kamenova, 2021) which suggest that parties like Alternative für Deutschland (Germany), and to some extent the Sweden Democrats and the Italian Lega Nord, deploy internal participation and even deliberative practices to strengthen their connections with citizens, challenging the generalization that all populist right-wing parties lack internal democratic mechanisms.

### Digital tools: participation, or mobilization?

The impact of digital tools on IPD remains a matter of considerable debate in literature. On the one hand, a body of research suggests that the integration of digital technology within new political parties facilitates increased membership participation ( Blasco, 2021; Correa *et al.*, 2021; Raniolo *et al.*, 2021; Vaccari, 2013). Conversely, an equally relevant stream of studies contests this perceived impact. Research by De Marco *et al.* (2019) indicates that newer parties do not exhibit substantial differences in representative-constituent interaction compared to their traditional counterparts. Furthermore, some evidence even suggests that oldest parties are currently more inclined to adopt online participation platforms for internal decision-making processes ( Sandri *et al.*, 2025). Supporting this skepticism, Bronet and Borge (2024) found that while new parties may display a slightly higher degree of digitalization, this is predominantly leveraged for resource mobilization rather than for significantly enhancing participatory mechanisms. This line of critique is further bolstered by the introduction of the pseudo-participation concept ( Biancalana & Vittori, 2021) to determine whether digital participation tools are employed strategically only to cultivate an impression among citizens and members that they influence party decision-making, reinforcing the argument that the transformative power of these technological innovations is often symbolic rather than substantive.

## Theoretical framework and hypotheses

The present study investigates the internal democracy of nascent Western European parties by employing a generational approach and a two-dimensional model of intra-party democracy (IPD), from which we derive our core hypotheses.

### Case selection: the generational approach of crisis parties

Delineating the precise corpus of young parties is the prerequisite step for this investigation. We adopt the generational approach proposed by Bronet and Borge (2024), which groups parties based on the shared contextual conditions (sociological, institutional, economic, and technological) surrounding their emergence. This framework delineates the recent boom of electorally successful new parties in the relatively homogeneous political landscape of Western Europe, primarily emerging from the 2008 economic crisis to the 2020 pandemic ( Bronet & Borge, 2024).

The authors propose moving beyond the terminology of "new" (and "digital") by adopting the concept of "crisis parties," which is defined as:

*“… the political parties formed in Western Europe in the 21st century, mainly from 2008 (economic crisis) to 2020 (pandemic), that have won at least one seat in the national or state parliament in at least one election, regardless of their ideology (from the extreme right to the radical left), the political issues they underscore or their provenance (i.e., whether they are genuinely/organizationally new or renewed). To fight against traditional parties that have lost legitimacy and to reconnect with citizens, most such parties use digital tools.” ( Bronet & Borge, 2024, p. 14–15)*

This framework offers a robust, geographically defined, large-N cohort for analysis, encompassing parties from various ideological spectrums and different digitalization levels, thus directly addressing the limitation of small-N bias and the over-reliance on leftist, highly digitalized cases identified in previous literature. While the generational approach may entail inherent challenges in selecting precise cut-off years and acknowledges potential intra-generational heterogeneity, its utility in capturing the boom of new parties and examining the collective influence of shared contextual factors on party organization remains high ( Bronet & Borge, 2024).

### Measuring internal democracy: AIPD and PIPD

With the cohort established, the second step is determining the metrics for assessing internal democracy. We adopt Von dem Berge and Poguntke’s (2017) two-dimensional model of IPD, which combines Assembly-based Intra-Party Democracy (AIPD) and Plebiscitary Intra-Party Democracy (PIPD), as the different modes of intra-party decision-making that the literature usually agrees with. These modes represent different logics—representative and direct democracy, respectively ( Brause & Poguntke, 2025b; Poguntke *et al.*, 2016). AIPD measures the inclusiveness of decision-making based on deliberation within party bodies (discussion and decision occurs together), whereas PIPD measures the extent to which all party members or supporters have a direct vote on party matters (discussion and decision are separated).We utilize the AIPD and PIPD indices developed by Von dem Berge and Poguntke (2017) using variables from the Political Parties Database Project (PPDB). These indices are established as suitable and robust tools for cross-national analysis ( Brause & Poguntke, 2025b). While we acknowledge that this model is based on formal party statutes, thus potentially creating a gap between formal and actual practice, its capacity for quantitative, cross-national comparison—particularly for assessing the plebiscitary character of crisis parties—renders it a sound analytical instrument. Furthermore, we will validate our PPDB-derived findings against the expert survey-based internal democracy measure provided by the Populism and Political Parties Expert Survey (POPPA database, Meijers & Zaslove, 2020; Meijers & Zaslove, 2021), which also allows us to introduce populism as an explanatory variable. Although this IPD measurement, based on experts’ opinions, does not distinguish between AIPD and PIPD, its operationalization relies on, among other studies, Poguntke *et al.*’s (2016) conceptual definition:

*“As defined in Poguntke *et al.* ( 2016, p. 670; see also Scarrow, 2015), intra-party democracy ‘maximizes the involvement of party members in the decisions that are central to a party’s political life, including program writing, and personnel selection and other intraorganizational decision-making’. Intra-democratic parties thus ‘are founded on principles of participation, competition, representation, responsiveness and transparency’ ( Rahat & Shapira, 2017, p. 88). What matters primarily is whether party members can influence decision making, internal debate is possible (and the party leadership does not rule this out) and procedures are inclusive of various factions and organizational layers within a party ( Meijers & Zaslove, 2021; Rahat & Shapira, 2017; Scarrow, 2015).”* ( Böhmelt *et al.*, 2022, pp. 1144)

### Theoretical derivation and hypotheses

The application of this framework to the crisis party cohort presents an opportunity to test assumptions about their internal organization. Previous studies, predominantly case-based and focused on left-leaning and highly digitalized parties, consistently suggest a plebiscitary model of intra-party participation for many new formations ( Barberà & Rodríguez-Teruel, 2020; Biancalana, 2016; Gerbaudo, 2019; Invernizzi-Accetti & Wolkenstein, 2017; Rodríguez-Teruel & Barberà, 2018). This expectation is grounded in the greater sensitivity of left-wing parties to internal democracy ( Pettitt, 2012; Poguntke *et al.*, 2016; Sandri *et al.*, 2025) and the potential for digital technology to enhance direct participation, thereby facilitating a PIPD mode ( Scarrow *et al.*, 2022a). Consequently, a plebiscitary mode of IPD is strongly anticipated among left-wing and highly digitalized crisis parties. Notwithstanding, the IPD structure of other crisis parties may also diverge from older party cohorts. Recent research indicates a potential trend toward greater internal democracy even in some new centrist ( Correa *et al.*, 2021) and right-wing parties. For instance, Alternative für Deutschland (a German far-right party) exhibits a notable degree of internal participation ( Kamenova, 2021). Hence, The Von dem Berge and Poguntke’s (2017) observation (based on older parties) that high PIPD often complements high AIPD may not hold true for the crisis party generation. Consequently, we hypothesize that the IPD of the crisis parties overall is more inclined toward a plebiscitary form than that of older cohorts, with ideology and digitalization serving as key explanatory variables.

This theoretical background leads to our first hypothesis, operationalized into two sub-hypotheses:

*H1a: Crisis parties—especially left-wing and highly digitalized—are expected to be more plebiscitary than older parties.*

*H1b: Older parties—especially on the left—are expected to be more assembly-based than crisis parties.*

Notwithstanding the expected plebiscitary nature of crisis parties, populism introduces a counter-theoretical dimension. For populist parties—a category frequently encompassing crisis parties— an initial hypothesis, based on their anti-elitist rhetoric, might predict a positive correlation between a high PIPD score and a low AIPD score ( Poguntke *et al.*, 2016). That is, their foundational ideational form (narrative) should theoretically favor greater internal democracy, particularly a plebiscitary structure. However, empirical evidence contradicts this expectation. Böhmelt *et al.* (2022), utilizing data from the POPPA, identified a negative correlation between a party's degree of populism and its internal democratic practices. This suggests that a party's populist intensity is inversely related to its level of internal democracy, leading to a more leader-centric structure. Nevertheless, Böhmelt *et al.* (2022) also noted a key ideological distinction, observing that left-wing populist parties tend to exhibit higher levels of internal democracy compared to their right-wing populist counterparts.

This leads to our second hypothesis:

*H2a: Populism mediates the relationship between crisis-party status and internal democracy.*

*H2b: Left-wing populist crisis parties are expected to be more democratic than right-wing populist ones.*

## Data and methods

The dataset was compiled from two distinct sources, each corresponding to one of the two stated hypotheses. Specifically, the Political Parties Database (PPDB) Round 2 ^1^ was utilized to address the requirements of the first hypothesis. Released in March 2022, the PPDB second round covers 288 parties in 51 countries, with 427 variables coded between 2016 and 2019. Consistent with the framework developed by Bronet and Borge (2024), the sample for this investigation comprised political parties operating within the homogeneous context of Western Europe ^2^. In operationalizing the concept, “crisis parties” were specifically delineated as those founded from the year 2006 onward that had subsequently attained a minimum of one electoral seat in either a national or state legislative body during at least one election. The selection criteria for identifying crisis parties utilized the 2008 global financial crisis and the 2020 COVID-19 pandemic as principal reference milestones. However, the emergence and trajectory of the crisis party phenomenon are neither instantaneous nor subject to abrupt termination. Consistent with the methodological approach of Bronet and Borge (2024), we have incorporated Western European parties established up to two years prior to the 2008 economic recession. This extension ensures that the analysis encompasses early-stage parties that structurally align with our definition of a crisis party. The temporal threshold for the inclusion of crisis parties was set at 2019, consistent with the final data point available in the PPDB Round 2 dataset. This specific cutoff is methodologically beneficial, as it immediately precedes the pandemic and the subsequent observed deceleration in new party formation.

PPDB Round 2 does not include any crisis parties from Finland, Sweden, and the United Kingdom; thus, we do not take into account the 23 older parties coded in our study to avoid biases, in line with Bronet and Borge (2024). Therefore, our sample comprised 29 crisis parties (some specific features of selected crisis parties are enumerated in Bronet & Borge, 2024, Appendix 2) and 69 older cohort parties from 13 countries. A total of 98 Western European parties. The parties (crisis and older, respectively) included in this study can be found in the shared dataset ( Bronet, 2025).

To conduct our analysis, we utilize the variables and coded data established by Bronet and Borge (2024). These key variables include the year of party foundation and cohort, region of Europe, left-center-right ideology, and the digitalization index. This previously validated index was constructed—similarly to the approach employed by González-Cacheda and Cancela Outeda (2024)—by incorporating eight PPDB variables from the second round, which are directly related to parties' website affordances. Critically, this is the same database used to analyze the IPD indices in the present study ^3^. Second, we added the IPD indices (AIPD index and PIPD index). The IPD scores of the political parties were calculated following Von dem Berge and Poguntke’s IPD model ( 2017) and kindly provided to us for this research by Brause and Poguntke (2025a) when they were not yet published ^4^. The characteristics and values of the new variables considered in this study are available in the shared dataset ( Bronet, 2025).

 In relation to the variables considered, a subset of parties lacks IPD scores. No calculations could be performed because the necessary data was not available in PPDB Round 2. Since three parties do not have AIPD values (all of them crisis) ^5^, for our AIPD analysis, we have considered 26 crisis parties from 13 countries, and all 69 older cohort parties from 13 countries. A total of 95 parties from 13 countries participated in the study. Moreover, the manifesto AIPD dimension (program component) has many missing data ( Brause & Poguntke, 2025b; Von dem Berge & Poguntke, 2017). To control for this effect, there is another AIPD index calculated without the manifesto dimension. While the differences were subject to scrutiny, the divergence between the AIPD index scores and the scores omitting the manifesto dimension was determined to be negligible. We also note that parties that do not have AIPD values do not have PIPD values. In addition, there are three parties that have AIPD values but do not have PIPD values (two crises and one older) ^6^. For the subsequent PIPD analysis, a sample comprising 24 crisis parties and 68 older cohort parties was utilized, drawn from 13 countries. A total of 92 parties from 13 countries. With respect to the digitalization index, Bronet and Borge (2024) reported that only three parties (all of them crisis) do not have PPDB data to calculate their digitalization scores ^7^ (all 69 older parties have data).

We consider that the obstacles to the available PPDB data we have just presented are minor and do not represent significant biases for our analysis. In fact, , the PPDB constitutes a relevant source of empirical data that can be leveraged to facilitate the comparative analysis of political parties and, thereby, identify prevailing trends ( Poguntke *et al.*, 2016) as González-Cacheda and Cancela Outeda's (2024) or Bronet and Borge’s (2024) recent works have proved.

To address the second hypothesis, and corroborate the IPD comparison between cohorts, data from the first wave of the Populism and Political Parties Expert Survey ( Meijers & Zaslove, 2020; Meijers & Zaslove, 2021) were utilized. This first wave was fielded in 2018 by country experts. This first wave was selected because its data collection year aligns with the coding period of PPDB round two and, critically, because the subsequent Wave 2 did not introduce new data regarding internal party democracy. The POPPA first wave measures the positions and attitudes of 250 parties on key characteristics related to populism, political style, party ideology, and party organization in 28 European countries. Analogous to the procedure applied for the PPDB, for our analysis we selected political parties from Western European countries and considered as crisis parties those founded from after 2006. The 2018 POPPA does not include any crisis parties from Finland, Sweden, and the United Kingdom, as happens in PPDB Round 1, but there is no crisis party from Norway and Portugal, countries that have crisis parties in the PPDB. Thus, we did not consider older parties' data from these five countries. Instead, the POPPA includes some crisis parties that the PPDB does not, such as La France Insoumise or Compromís (Spain). In total, we observed 109 Western European parties from 11 countries, 40 crisis, and 69 older. Consult our shared dataset ( Bronet, 2025) to see the POPPA parties considered, crisis, and older. To select, classify, and compare these parties, we added some of the same variables that we also created in the PPDB: cohort (based on the party foundation year), region of Europe, and left-center-right ideology (based on the party ideology numerical variable of POPPA ^8^ Subsequently, attention was directed toward two key POPPA variables: intradem ^9^ and populism_cfa_rescaled ^10^. All parties in our sample have populism values and only four parties do not have intradem values ^11^.

The methodology employed for both datasets (PPDB and POPPA) followed a two-step approach to investigate differences in IPD: preliminary analysis, and explanatory modeling. First, a comparative analysis of the defined cohorts was conducted using descriptive statistics and bivariate analyses. This stage provided an initial observation and comparison of the characteristics and relationships between the key variables within each dataset. Following the preliminary analysis, multiple linear regression models were constructed to determine the independent effects of the variables on IPD. The selection of explanatory variables for each model was based on the specific focus of the dataset: the PPDB model included Cohort, Ideology, and Digitalization, while the POPPA model included Cohort, Ideology, and Populism. These models were utilized to identify the variables that significantly account for the observed variance in IPD.

We checked the normal distribution of the numerical dependent and independent variables using the Shapiro-Wilk W test and multicollinearity between the independent variables with the VIF measures. We also assessed the degree of alignment between the POPPA manner of measuring internal democracy and the Von dem Berge and Poguntke IPD. Thus, we added the POPPA intradem values to concurrent parties in our PPDB sample, and Spearman’s rank correlation coefficient was calculated between the variables. The first correlation, between the AIPD index and the POPPA internal democracy scale, yielded a coefficient of *ρ* = 0.298 (p = 0.008), while the second, comparing the PIPD index to the same POPPA scale, was slightly higher ( *ρ* = 0.328, p = 0.004). Although the correlations were moderate, both were statistically significant, suggesting a consistent association between the measures. This indicates that despite differences in operationalization, the indicators tend to capture a similar underlying pattern.

## Empirical analysis/results

The first hypothesis argues that the crisis parties are more plebiscitary than older cohorts, especially if the crisis parties are on the left and highly digitalized (H1a). In contrast, the older cohort’s parties, especially the old left-wing parties, have higher levels of assembly-based IPD than the crisis parties (H1b). Being more plebiscitary than the older cohort’s parties means that the crisis parties have higher PIPD scores than the older parties, the crisis parties have fewer gaps between AIPD and PIPD values (because it could be that the AIPD scores of these parties were lower) than the older parties and/or the crisis parties have PIPD values over AIPD scores. In light of this analysis, the study will also ascertain the extent to which these political parties possess an essentially plebiscitary character. This observation further facilitates an assessment of whether crisis parties exhibit higher levels of internal democracy compared to older parties, a comparison achieved through the analysis of their AIPD and PIPD scores, although the effect of plebiscitary decision-making on overall democratic quality remains a normative and contested debate ( Brause & Poguntke, 2025b).

**Figure 1.**
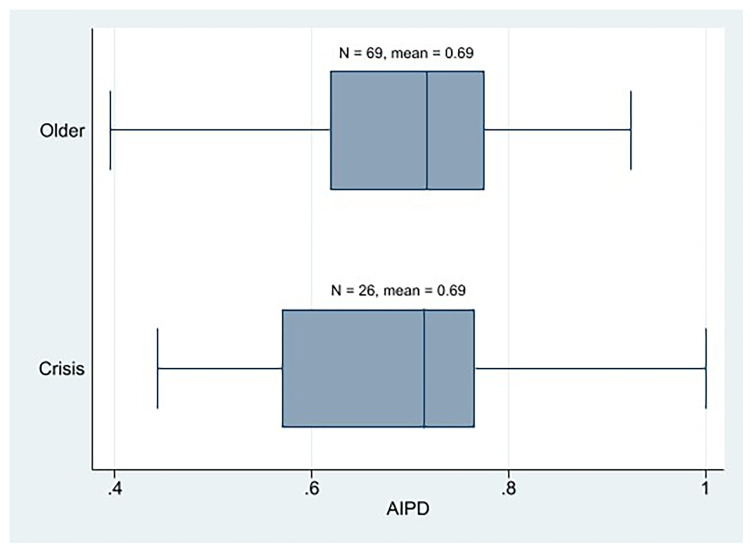
Western European parties AIPD by cohort.

**Figure 2.**
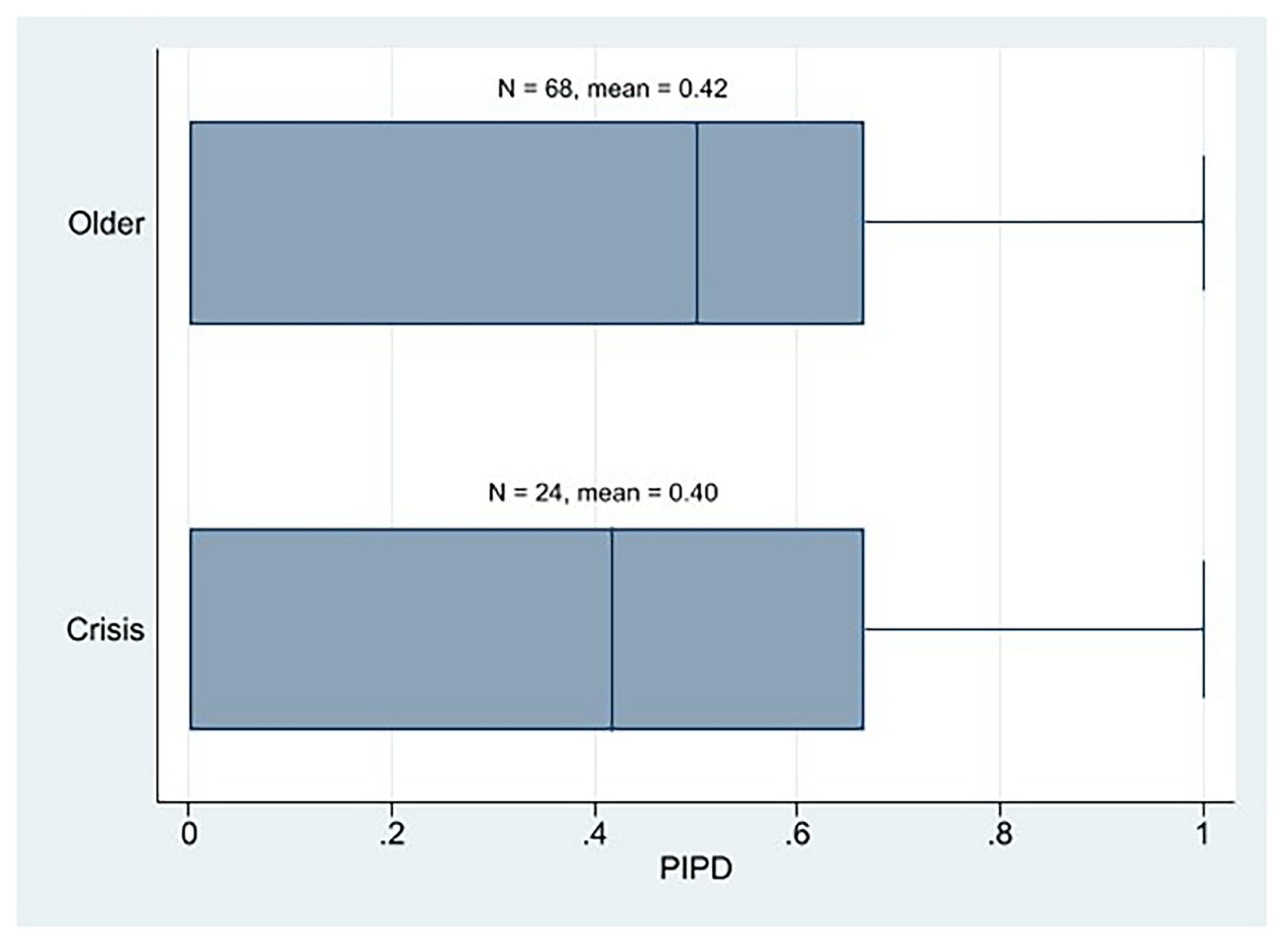
Western European parties PIPD by cohort.

At the descriptive level, we observe that the AIPD mean of the crisis parties is the same as that of the older Western European parties ( Figure 1), and the PIPD mean of the crisis parties is slightly lower than that of the older parties ( Figure 2). In addition, the AIPD mean of the crisis parties was clearly higher than that of its PIPD. Therefore, at first glance, we might not state that the crisis parties are more plebiscitary than the older parties, nor are the crisis parties essentially plebiscitary. It cannot be conclusively determined that the crisis parties exhibit either a greater or lesser degree of democracy than the established parties, as evidenced by the similarity in their respective IPD mean scores (AIPD and PIPD).

 More specifically, only five crisis parties (out of 26) have higher PIPD than AIPD values ^12^. In all these cases, the AIPD scores were also considerably high (from 0.57 to 0.78). Von dem Berge and Poguntke (2017) pointed out that when parties have high levels of AIPD, high levels of PIPD do not necessarily lead to empowering elites. Only three (out of 26) scored 1 in the PIPD index ^13^. Some new parties that literature usually takes as references for observing their internal democracy, such as Movimento 5 Stelle in Italy or Alternativet in Denmark, have higher AIPD scores than PIPD’s. In fact, the only frequently cited case with a higher PIPD than AIPD was Podemos in Spain. Hence, it is not evident that a defining characteristic of crisis parties is their inherently plebiscitary organizational nature.

Regarding older parties, 15 out of 69 older parties had higher PIPD than AIPD values and 10 scored 1 in the PIPD index. Both figures represent slightly higher rates than those of the crisis parties. Thus, there is a lower proportion of plebiscitary parties among the crisis parties than among the older cohort’s parties.

**Figure 3.**
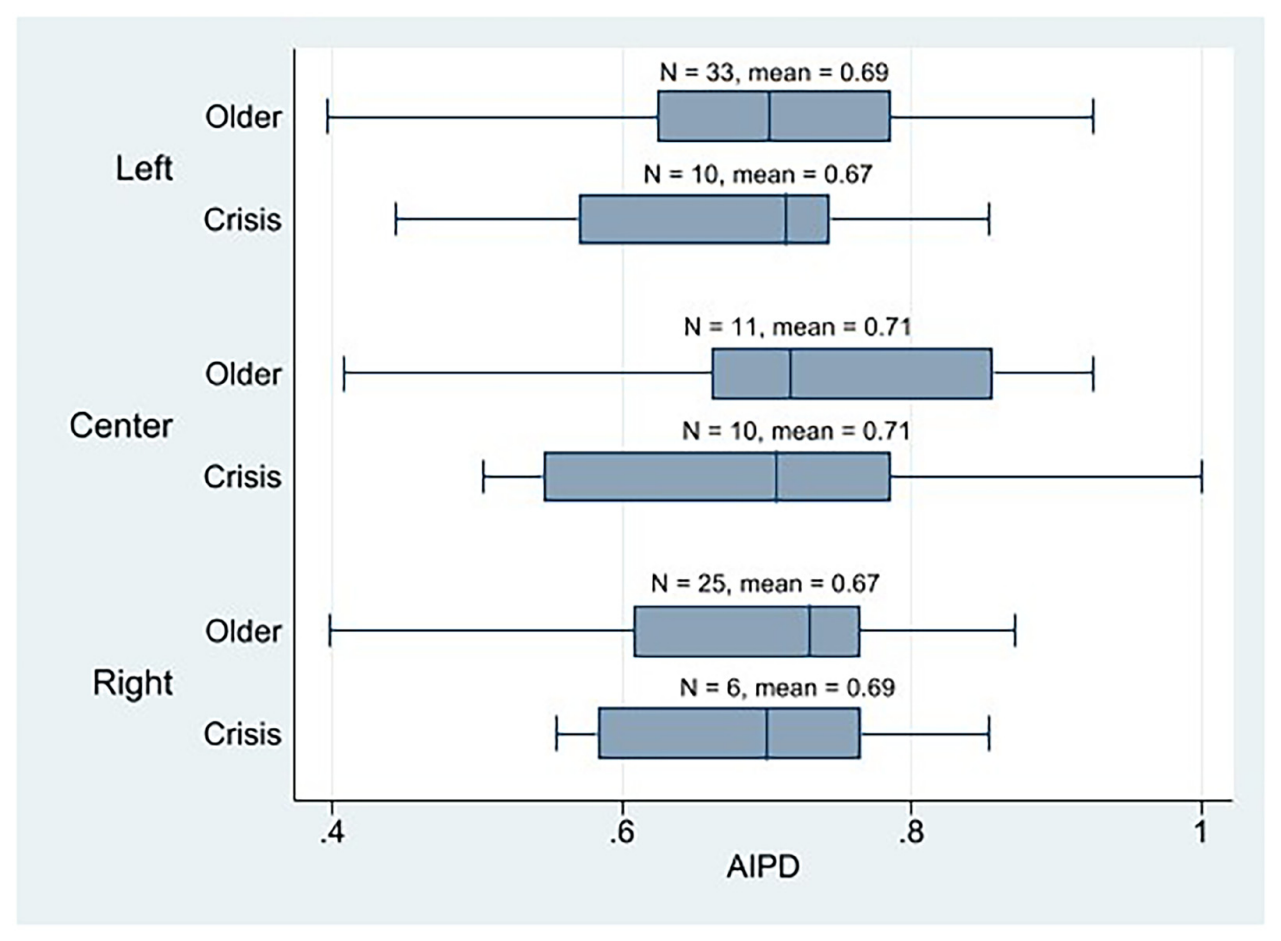
Western European parties AIPD by cohort and ideology.

**Figure 4.**
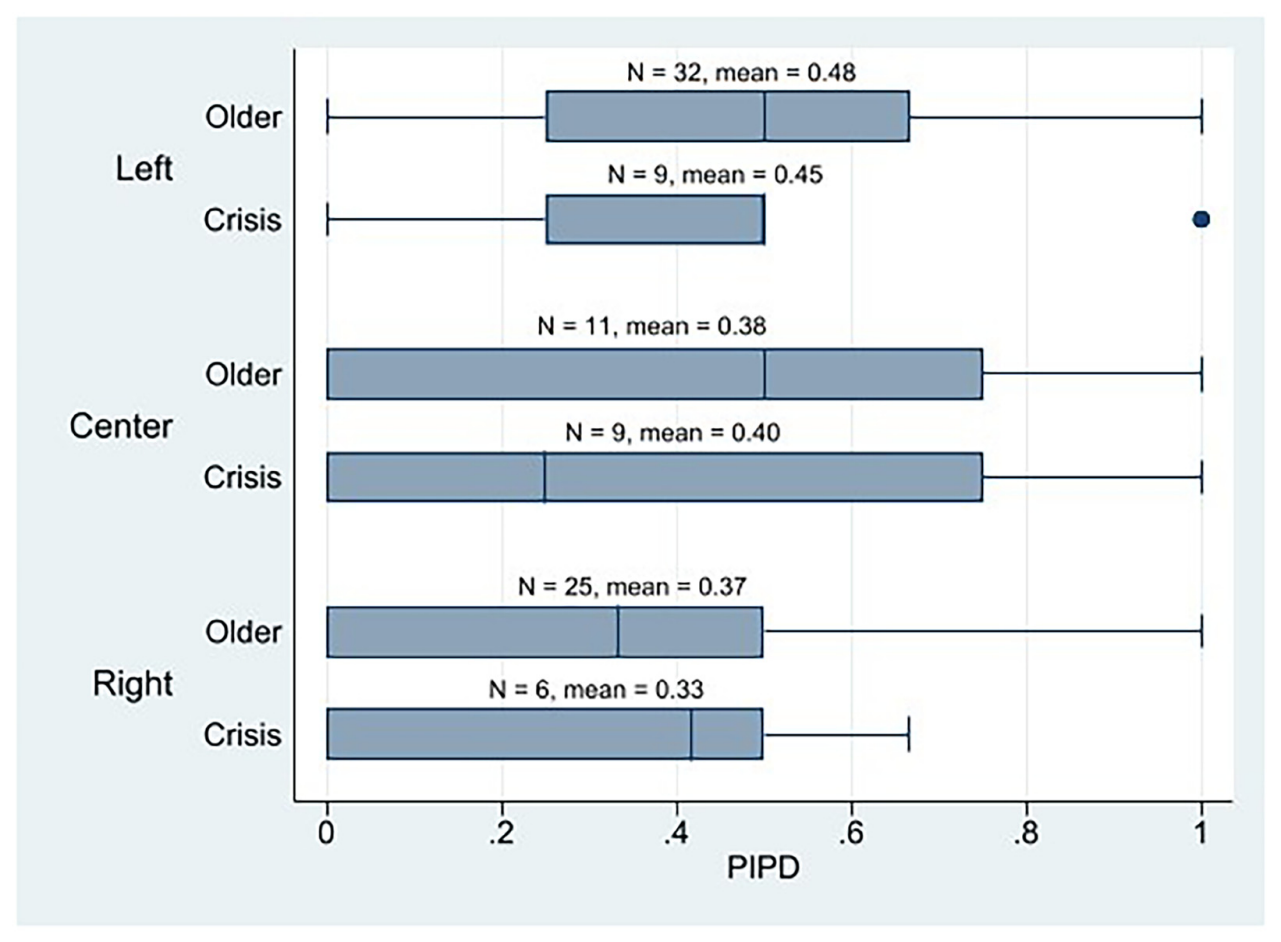
Western European parties PIPD by cohort and ideology.

 With respect to ideological alignment, statistical analysis reveals minimal variance in the mean AIPD observed among crisis parties, with the left parties surprisingly scoring the lowest and the center parties the highest ( Figure 3). Instead, left-crisis parties obtain the highest PIPD mean, followed by the center, and ending with the right parties ( Figure 4).

Therefore, by considering only the mean, we might say that the parties from the left wing would be the most plebiscitary parties among the crisis parties because they have the highest PIPD mean, and the difference between PIPD and AIPD exhibits the closest proximity. Yet, as depicted in the PIPD boxplot ( Figure 4), this observation is primarily attributable to the presence of two left-wing parties that registered a PIPD score of 1. No other left-crisis party scored above 0.5. Instead, there are four center parties that score over 0.5, in PIPD. We also observe that three out of the five crisis parties that have higher PIPD than AIPD values are center parties, and two are left ones. And two of the three crisis parties that score 1 in the PIPD index are left parties, and one is center. Thus, although their PIPD mean is slightly lower than that of the left-wing parties, the discernible representation and influence of centrists or liberals within highly plebiscitary crisis parties constitutes a significant observation. The AIPD mean of the center crisis parties (and the older’s too) is also notable. Taken together, these results suggest that center crisis parties exhibit a high degree of internal democracy. It is evident that right-wing crisis parties exhibit the lowest levels of plebiscitary participation (as quantified by the PIPD scores). Generally, however, crisis parties utilize the direct vote of their entire membership as a decision-making mechanism, thereby complementing their already robust representative devices.

Consequently, it would be difficult to justify the assertion that the crisis parties on the left—most of which profess a commitment to enhancing internal democracy to restore legitimacy—are essentially plebiscitary, with the exception of specific, limited cases ^14^. The observation that the left-wing parties exhibit the highest mean PIPD score, alongside the narrowest differential between the PIPD and AIPD mean scores within the crisis parties, does not necessarily imply an inherently plebiscitary organizational character. This interpretation is mitigated by the fact that the mean AIPD score for the left-wing crisis parties is conspicuously higher than their PIPD mean. Moreover, the PIPD mean of the older left-wing parties is even higher than that of the left-wing crisis parties ( Figure 4). A similar pattern emerges when comparing the mean PIPD scores between highly digitalized ^15^ left-wing crisis parties and highly digitalized left-wing older cohort parties ( Table 1). Specifically, within the set of the most highly digitalized parties, a statistically significant positive correlation was observed between left-wing political orientation and PIPD (p = 0.041). However, it is important to note that the highest mean PIPD score was recorded by the highly digitalized center-based crisis parties.

**Table 1.** PIPD of the highly digitalized parties by ideology and cohort.

Ideology	N (Crisis)	Mean (Crisis)	SD (Crisis)	N (Older)	Mean (Older)	SD (Older)
Left	7	0.476	0.413	16	0.521	0.321
Centre	6	0.569	0.370	7	0.321	0.345
Right	3	0.333	0.289	12	0.257	0.240

*Note: PIPD stands for Plebiscitary Intra-Party Democracy. Cohort: "Crisis" = Western European parties founded from 2006 onwards; "Older" = Western European parties founded before 2006. Ideology is coded as Left (1), Centre (2), Right (3).*

Regarding the second component of the first hypothesis (H1b), which posits that older cohort's parties, particularly those on the left-wing, would exhibit higher levels of assembly-based intra-party democracy (AIPD) compared to crisis parties, preliminary analysis was conducted. As previously noted, the aggregate AIPD mean for the older party cohort did not differ from that of the crisis parties. However, a more nuanced observation emerges when comparing specific party sub-groups: the older left-wing parties demonstrated a marginally higher AIPD mean than their counterparts in the crisis party cohort; nevertheless, the magnitude of this difference was minimal (as visually represented in Figure 3). Furthermore, it is noteworthy that the center parties—encompassing both the older and crisis cohorts—obtained the highest AIPD scores across the analyzed spectrum. These descriptive results should be interpreted with caution, as the low number of data points within each segmented group may compromise their statistical robustness.

Inferential analysis was performed using two multiple regression analyses to determine the predictive capacity of the specified independent variables regarding the level and form of IPD among political parties in Western Europe. This approach served to statistically substantiate the relationships tentatively identified through the preceding descriptive investigation. In the first regression, the dependent variable is the AIPD index ( Table 2), and in the second, the PIPD index ( Table 3). The explanatory variables are cohort (being a crisis party or an older party), ideology, and the level of digitalization (numerical -0 to 1-). The metrical variables follow a normal distribution ^16^ and there is no multicollinearity ^17^. Due to the limited sample size, especially for crisis parties (n = 26 for AIPD and n = 24 for PIPD), a bootstrapping methodology was employed to enhance the robustness of the estimates. It is a resampling method that relaxes distributional assumptions and is well-suited for small samples. Below is a summary of the regression analysis results.

**Table 2.** Linear regression predicting AIPD (PPDB). Bootstrapped standard errors (1,000 replications).

Variable	Coefficient	Std. Error	z	p-value	95% CI
Cohort	-0.023	0.030	-0.76	0.448	[-0.082, 0.036]
Digitalization	0.291	0.087	3.33	0.001 ***	[0.120, 0.462]
Ideology	-0.0004	0.014	-0.03	0.978	[-0.028, 0.027]
Constant	0.563	0.051	11.08	0.000 ***	[0.464, 0.663]

**Model statistics:** N = 93       R² = 0.133       Adj. R² = 0.104       Root MSE = 0.124       Prob > χ² = 0.0076 **Notes:**     •   Dependent variable: *AIPD* (Assembly-based Intra-Party Democracy index).     •   Standard errors are based on 1,000 bootstrap replications.     •   ***p < 0.01.

**Table 3.** Linear regression predicting internal PIPD (PPDB). Bootstrapped standard errors (1,000 replications).

Variable	Coefficient	Std. Error	z	p-value	95% CI
Cohort	-0.045	0.077	-0.59	0.556	[-0.196, 0.106]
Digitalization	0.335	0.172	1.94	0.052 *	[-0.003, 0.672]
Ideology	-0.058	0.039	-1.48	0.138	[-0.135, 0.019]
Constant	0.386	0.110	3.50	0.000 ***	[0.170, 0.602]

**Model statistics:** N = 91      R² = 0.052      Adj. R² = 0.019      Root MSE = 0.337      Prob > χ² = 0.119 **Notes:**     •   Dependent variable: *PIPD* (Plebiscitary Intra-Party Democracy index).     •   Standard errors are based on 1,000 bootstrap replications.     •   *p < 0.1, ***p < 0.01.

Digitalization was identified as a significant predictor for both the AIPD and PIPD indices. Consequently, the degree of digitalization is positively correlated with IPD levels. Specifically, a higher level of digitalization in a Western European political party corresponds to greater internal democracy, independent of its formation cohort (i.e., whether it is a crisis-formed or older party) or ideological stance (left-wing or right-wing). Other variables incorporated into our analytical model—namely, party cohort and ideology—did not exert a significant influence on the magnitude or form of intra-party democracy. This inferential analysis thus confirms the descriptive observation that IPD does not significantly vary based on the party's cohort or ideology. Therefore, crisis parties are not demonstrably more plebiscitary (nor more democratic) than older parties, and conversely, older parties are not characterized by a greater degree of assembly-based democracy compared to crisis parties.

Our second hypothesis proposes that populism mediates the relationship between crisis-party status and internal democracy (H2a). Furthermore, we hypothesize that left-wing populist crisis parties will exhibit higher levels of internal democracy than their right-wing populist counterparts (H2b).

**Figure 5.**
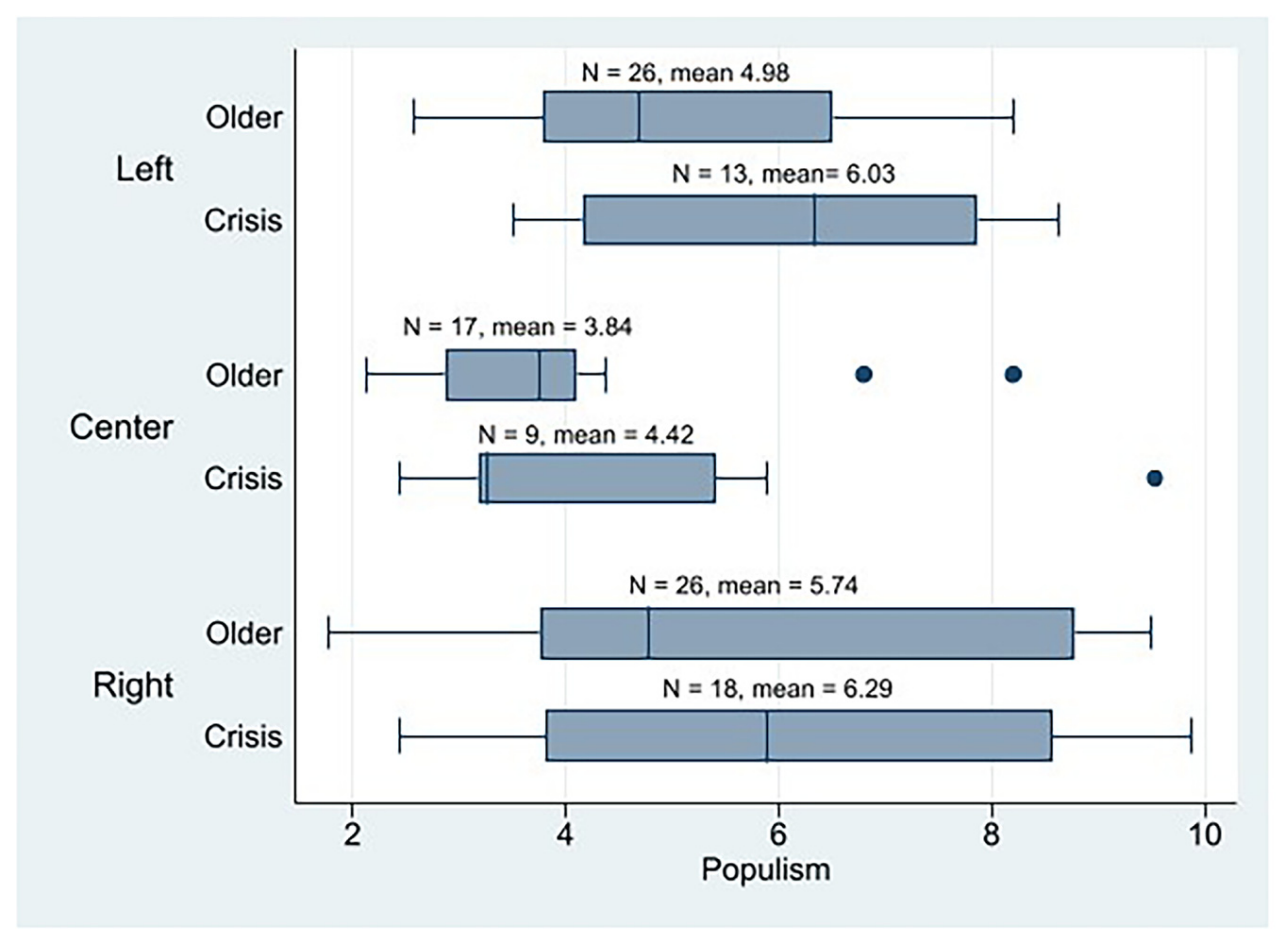
Western European parties populism by cohort and ideology.

At the descriptive level, we note that the crisis parties in general are more populist (n = 40, mean = 5.79) than the older cohorts (n = 69, mean = 4.99). In Figure 5, we can observe that the crisis parties of all three ideologies are more populist than their older counterparts by considering their means. Moreover, 23 out of 40 crisis parties scored 5 or more on the populism scale (58%), which could be referred to as populists, while 24 out of 69 older parties do (35%). Accordingly, the prevalence of populist parties is significantly greater within the cohort of crisis parties compared to the older parties. Therefore, the crisis parties would be more populist than the older cohort’s parties, as Katz (2021) points out when discussing the increase in anti-party-system parties. As shown in Figure 5, ideology appears to be a relevant factor that explains populism as well (being the center parties the least populist).

**Figure 6.**
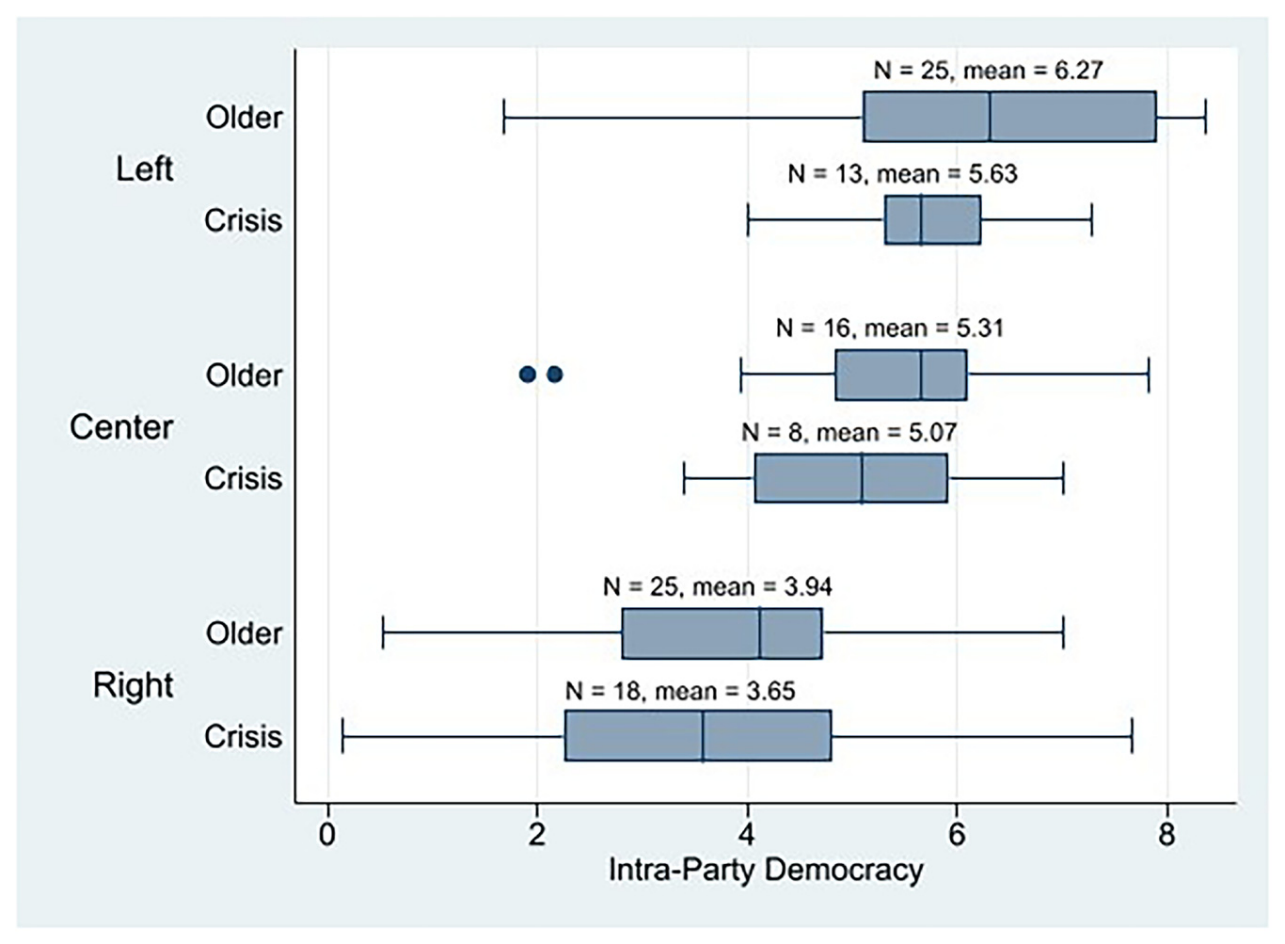
Western European parties IPD by cohort and ideology.

Thus, if intra-party democracy is negatively correlated with populism ( Böhmelt *et al.*, 2022) and the crisis parties are more populist than the older cohort parties, the crisis parties should be less internally democratic than the older parties. The intra-party democracy means demonstrate that the crisis parties’ mean was slightly lower (n = 39, mean = 4.60) than that of the older parties (n = 66, mean = 5.15). Figure 6 illustrates how ideology intersects with cohorts (impacted by populism) to influence internal democracy. Left-wing old parties are posited to exhibit the highest levels of internal democracy, sequentially followed by the left-leaning crisis parties. The right crisis parties are observed to demonstrate the lowest level of internal democracy. The IPD mean of the crisis parties across all three ideologies was lower than that of their older counterparts.

If we consider only the populist ^18^ crisis parties, we observe that the left parties (n = 8, mean = 5.49) are more democratic than the center (n = 3, mean = 4.64) and right parties (n = 12, mean = 2.80). As with the descriptive analysis of the PPDB dataset, these results should be interpreted cautiously due to the limited size of certain crisis party subgroups.

Consistent with the methodology applied for the first hypothesis, a multiple regression analysis was conducted using the POPPA sample ( Table 4). This analysis was performed to investigate the influence of the independent variables on internal democracy and to validate the initial descriptive observations. The dependent variable is the internal democracy index (numerical -0 to 10-), and the explanatory variables are cohort (dummy in which the reference category is older party), ideology (dummy in which the reference category is center), and level of populism (numerical -0 to 10-). The internal democracy index followed a normal distribution ^19^, but the populism level did not ^20^, and there was no multicollinearity ^21^. Although the distribution of the populism variable is not strictly normal, normality is not required for independent variables in linear regression. What matters is the distribution of the residuals, which is handled in this case using bootstrapped standard errors. This approach avoids reliance on parametric assumptions including normality. In addition, the populism variable is rescaled and used in its form by Böhmelt *et al.* (2022). Table 4 provides a summary of the regression analysis.

**Table 4.** Linear regression predicting internal democracy (POPPA). Bootstrapped standard errors (1,000 replications).

Variable	Coefficient	Std. Error	z	p-value	95% CI
Crisis parties	-0.032	0.258	-0.13	0.900	[-0.537, 0.473]
Left (vs. Centre) ideology	1.445	0.319	4.53	0.000 ***	[0.820, 2.071]
Right (vs. Centre) ideology	-0.425	0.318	-1.34	0.181	[-1.048, 0.198]
Populism	-0.497	0.060	-8.29	0.000 ***	[-0.614, -0.379]
Constant	7.251	0.332	21.82	0.000 ***	[6.600, 7.903]

**Model statistics:** N = 105       R² = 0.591       Adj. R² = 0.574       Root MSE = 1.239       Prob > χ² = 0.000 **Notes:**     •   Dependent variable: *intradem* (internal democracy index).     •   Reference category for ideology: Centre.     •   ***p < 0.01.     •   Standard errors based on 1,000 bootstrap replications.

With our sample of only Western European parties, one can observe what Böhmelt *et al.* (2022) already found for all parties included in the POPPA, in the sense that ideology and populism affect internal democracy. There is a positive correlation between being a left-wing party and internal democracy, as well as a negative correlation between populism and internal democracy. Thus, we can confirm what is observed with the descriptive statistics, populism, and ideology (being a left party) are significant variables that may explain intra-party democracy with very good capacity (high R²) in our model. Instead, the cohort does not seem to directly influence IPD. Nevertheless, the cohort affects intra-party democracy to the extent that crisis parties are more populist than older parties. Consequently, populism mediates the relationship between crisis-party status and internal democracy (H2a).

Regarding the populist crisis parties, we find a significant relationship between ideology and intra-party democracy IPD (p = 0.000). This finding supports the descriptive analyses relevant to the second part of our second hypothesis (H2b), namely, that left-wing populist crisis parties exhibit greater democratic characteristics than their right-wing populist counterparts.

## Discussion and conclusions

The operationalization of the datasets within our theoretical framework enables an empirical analysis that mitigates the biases present in the most relevant prior literature—namely the limited number of cases, and its concentration on left-leaning and highly digitalized parties. Consequently, our findings, based on a large-N sample, offer a more generalizable platform to systematically evaluate and contrast the conclusions typically drawn from high-digitalization and left-wing focused case studies by incorporating the underrepresented new right-wing and less digitalized Western European parties.

Bronet and Borge (2024) developed a generational approach to delineate the phenomenon of the boom of electorally successful new political parties in Western Europe over the past two decades. This conceptualization facilitated the establishment of the study's cohorts (i.e., crisis parties and older parties). The measurement of Intra-Party Democracy (IPD) draws on the model proposed by Von dem Berge and Poguntke (2017), which distinguishes between assembly-based and plebiscitary forms of IPD. Their IPD model, operationalized using PPDB data, is deemed suitable for the present research, enabling the findings to be situated within the existing literature.

Data for analyzing the IPD of crisis parties, facilitating comparative analysis both among them and with older parties, were sourced from the PPDB and the POPPA. Indeed, POPPA proved valuable for validating results obtained from the PPDB and for introducing populism as an explanatory variable for IPD. Crucially, POPPA Wave 1 was coded contemporaneously with PPDB Round 2 and encompasses a larger sample of crisis parties than the PPDB, thereby increasing the study's N. Although the POPPA measure of internal democracy does not explicitly differentiate between AIPD and PIPD, it is both theoretically and operationally well-aligned with the Von dem Berge and Poguntke’s IPD model.

Our work underscores the necessity of sustaining, updating, and enhancing cross-national data initiatives, such as the PPDB and POPPA. Continued investment in these projects is essential for accumulating the high-quality, comparative data required for rigorous quantitative research on political parties.

Drawing upon our theoretical framework, datasets, and methodology, our empirical analysis yields several salient findings that challenge prior assumptions.

### Divergence from plebiscitary expectations

Contrary to prior research—which led to the formulation of our Hypothesis 1a (H1a)—crisis parties are neither more plebiscitary than older Western European parties nor inherently plebiscitary. The expectation that crisis parties—particularly those on the left and highly digitalized—would exhibit a more plebiscitary intra-party democracy (PIPD) is not empirically supported. In fact, a lower incidence of highly plebiscitary parties was observed within the crisis party cohort compared to the older parties. Specifically, left-wing crisis parties, despite often emphasizing enhanced internal democracy to bolster legitimacy, are not found to be essentially plebiscitary, except in highly specific instances. Paradoxically, the older left-wing parties emerged as the most plebiscitary group. While left-wing parties within the crisis cohort do appear to be the most plebiscitary among crisis parties, the contribution of center crisis parties to the highly plebiscitary crisis party group is notable. Furthermore, within the set of highly digitalized political parties, the most plebiscitary organizations are identified as center crisis parties, rather than as left-wing crisis parties. These are subsequently followed by older, highly digitalized left-wing parties. This observation directly contrasts with previous literature, which often suggests, mainly based on case studies, that the internal democracy of highly digitalized left-wing crisis parties adopts a plebiscitary form ( Barberà & Rodríguez-Teruel, 2020; Biancalana, 2016; Gerbaudo, 2019; Invernizzi-Accetti & Wolkenstein, 2017; Rodríguez-Teruel & Barberà, 2018). Notably, Podemos (Spain) is the only frequently cited case whose PIPD score exceeds its assembly-based intra-party democracy (AIPD) score. It is crucial to acknowledge that case studies may reveal detailed contextual factors—not captured by the PPDB database, which is primarily based on the normative dimension (party statutes)—that influence democratic practice.

### Refutation of assembly-based assumptions

The findings also indicate that parties of the older cohort do not exhibit higher levels of assembly-based IPD (AIPD) than crisis parties. The assumption that older parties—particularly those on the left—would be more assembly-based than crisis parties (H1b) is similarly refuted. The older left-wing parties do not constitute the most representative party group concerning assembly-based intra-party democracy (AIPD). Instead, center parties (both crisis and older) register the highest scores in AIPD. However, these results require a degree of cautious interpretation, given the small sample size in certain subgroups of the crisis party cohort.

### Populism and internal democratic deficits

Our study confirms that crisis parties exhibit higher levels of populism compared to the older cohort parties in Western Europe, consistent with observations regarding the rise of anti-party-system parties ( Katz, 2021). We observe that crisis parties across all three ideologies (left, center, and right) are more populist than their older counterparts. Moreover, the proportion of identified populist parties is greater within the crisis party group than among older parties. Given the established negative correlation between populism and IPD ( Böhmelt *et al.*, 2022), we should expect that crisis parties would thus be less internally democratic than older cohorts. Overall, this expectation is generally met: crisis parties are, on average, slightly less internally democratic than older parties. Our findings strongly indicate that populism serves as a significant mediator in the dynamics linking a party's crisis status and the observed level of internal democratic practice (H2a). This underscores the theoretical importance of populism in structuring intra-party politics. An analysis across the ideological spectrum of the designated 'crisis cohort' reveals that left-wing crisis parties emerge as the most internally democratic group, although they remain less internally democratic than their older left-wing counterparts. When the analysis is restricted solely to populist crisis parties, left-wing populist crisis parties are also found to be more democratic than both center and right populist crisis parties (H2b). It must be noted that these findings should be considered with caution, as segmentation of the sample results in subgroups with few observations.

The analysis of potential determinants influencing IPD focused on cohort, ideology, digitalization, and populism. Our findings indicate that, only when analyzing POPPA, ideology constitutes an element that affects intra-party democracy. Furthermore, digitalization demonstrates a robust impact on IPD (affecting both AIPD and PIPD), suggesting a positive correlation: increased digitalization in Western European parties corresponds to higher internal democracy. Therefore, digitalization is seen as a good condition or possibility of improving internal democracy. Populism also exerts a clear and significant influence. In contrast, the cohort itself (i.e., being a crisis party versus an older party) does not appear to directly influence IPD. Instead, cohort affects internal democracy indirectly in that crisis parties are generally more populist than older parties, which subsequently leads to lower levels of internal democracy.

In conclusion, our empirical analysis does not support the hypothesis that a party's status as a crisis-driven entity, even one characterized by a high degree of digitalization and a left-wing orientation, significantly contributes to a more plebiscitary internal democracy. The underlying factor is that crisis parties exhibit a higher degree of populism compared to older-cohort parties, which, in turn, appears to negatively correlate with and potentially restrict their internal democratic processes. Consequently, this relationship serves to attenuate their anticipated theoretical potential for PIPD. We suggest that institutionalization within crisis parties may also represent a potential mechanism influencing whether their intra-party democratic patterns converge toward those characteristics of older political parties. Ultimately, digitalization exhibits a strong predictive power concerning intra-party democracy. Consequently, digitalization enables political parties to expand the participation mechanisms available to their rank-and-file members. This contribution enhances the conceptualization of digitalization as a democratizing impetus within party organizations.

This study provides a fundamental empirical analysis of crisis party dynamics. To further enhance and complement our findings, we identify a key opportunity for continued investigation: the evolution of IPD within these parties. It is of significant academic interest to determine whether the ongoing institutionalization process among these crisis-born political organizations is leading to a quantifiable shift towards either a less democratic structure or a more plebiscitary model of internal governance. Furthermore, future research should explore the potential correlation between temporal variations in IPD and changes in electoral support. Specifically, we hypothesize that a decline in national parliamentary representation (loss of seats) among these crisis parties may be directly associated with their degree of institutionalization and a corresponding reduction in IPD. The forthcoming third round of the PPDB is anticipated to furnish the necessary data to address these specific research questions, thereby offering a more comprehensive understanding of the interplay between internal party structure and electoral outcomes. The scope of this research could also be further enhanced by incorporating data gathered through surveys, focus groups, and semi-structured interviews with members and leaders of select crisis parties. This approach would facilitate the acquisition of their specific views and perceptions regarding the temporal evolution of their internal democracy, thus allowing for a crucial contrast with the fundamentally normative dimension provided by the PPDB.

## Ethics and consent

Ethical approval and consent were not required.

## Data Availability

All data and materials are available in the Open Science Framework. Root data **DOI:** https://doi.org/doi:10.7910/DVN/0JVUM8 ( Scarrow *et al.*, 2022b). Political Party Database Round 2 v4 (First Public Version). Harvard Dataverse. Data are available under the terms of the Creative Commons Zero “No rights reserved” data waiver (CC0 1.0 Public domain dedication). **DOI:** https://doi.org/doi:10.7910/DVN/GKFIV3 ( Brause & Poguntke, 2025a). Intra-Party Democracy Indices (V1) Based on PPDD Round 2. Harvard Dataverse. Data are available under the terms of the Creative Commons Zero “No rights reserved” data waiver (CC0 1.0 Public domain dedication). **DOI:** https://doi.org/doi:10.7910/DVN/RMQREQ ( Zaslove *et al.*, 2024). Populism and Political Parties Expert Survey 2023 (POPPA). Harvard Dataverse. Data are available under the terms of the Creative Commons Zero “No rights reserved” data waiver (CC0 1.0 Public domain dedication). New data Repository: CORA.Research Data Repository. https://doi.org/10.34810/data2523 ( Bronet, 2025). This dataset contains the following files: PPDB_R2_Crisis Parties. (PPDB Round 2 of the political parties from the Western European countries that include Crisis Parties with these new variables added at the end: year of party foundation, cohort -crisis/older party-, region of Europe, left-center-right ideology, digitalization index, aipd, pipd, oipd, aipd_wm, and POPPAintradem). Original data are in STATA format. Poppa_W1_Crisis Parties. (POPPA Wave 1 of the political parties from the Western European countries that include Crisis Parties with these new variables added at the end: cohort -crisis/older party-, region of Europe, left-center-right ideology). Original data are in STATA format. Data are available under the terms of the Creative Commons Zero "No rights reserved" data waiver (CC0 1.0 Public domain dedication).

## Footnotes

^1^ This dataset was observed to possess some limitations. A recognized constraint of the PPDB—constructed through expert coder collaboration—is the presence of missing data ( Scarrow *et al.*, 2022b). Specifically, it was observed that a significant number of extra-parliamentary political parties are not cataloged within the PPDB. However, a critical selection criterion for inclusion in the present study's crisis party cohort necessitates electoral success, defined as securing at least one seat in a national or state parliament in at least one election. A further limitation is the omission of specific crisis parties that achieved parliamentary representation subsequent to the PPDB Round 2 data collection. This constraint has been previously documented (e.g., Bronet & Borge, 2024), affecting parties such as La France Insoumise and Vox. Nevertheless, it is important to note that the second round comprehensively encompasses the majority of parties represented in national parliaments at the time of its coding.

^2^ Austria, Belgium, Ireland, Spain, Portugal, United Kingdom, Switzerland, France, Denmark, Finland, Germany, Italy, Netherlands, Norway, Greece, Sweden.

^3^ While acknowledging more recent and advanced digitalization studies, such as that by Sandri *et al.* (2025), the PPDB dataset is preferred due to its broader cross-national coverage and its concurrent temporal coding scheme.

^4^ The PPDB variables selected for the construction of the IPD index are specified in Appendixes A.2.1 and A.2.2 of Brause and Poguntke (2025b).

^5^ Independents4Change and Solidarity-People Before Profit, both left-wing parties from Ireland, and Party for Freedom, a right party from the Netherlands.

^6^ Liberal Alliance (center party from Denmark), DENK (left party from the Netherlands) and Ecologist Party "The Greens" (left party from Portugal).

^7^ Parti Populaire (right party from Belgium), the Liberal Alliance (center party from Denmark) and Independents4Change (left party from Ireland).

^8^ >= 4 left, >4 & <6 center, and >= 6 right on the “lroverall” variable scale.

^9^ POPPA item: “Some political parties practice more intra-party democracy than others (i.e., party members’ play a role in decision-making, there is room for internal debate, decision-making is inclusive of various factions and organizational layers within the party)”. Experts rate parties on a scale from 0 to 10, with 10 standing for a very high level of intra-party democracy (0 stands for no intra-party democracy at all).

^10^ It captures the degree of populism for each political party in the data as taken from POPPA. This variable is a weighted arithmetic mean of its five subcomponents: “manichean”, "indivisible", “generalwill”, “peoplecentrism”, and “antielitism”. It ranges between 0 (a party is not at all populist) and 10 (a party is very populist).

^11^ DENK (Netherlands), as a crisis party, and Canary Coalition (Spain), Federal Democratic Union of Switzerland (Switzerland) and Swiss Party of Labour (Switzerland), as old parties. Two left-wing parties, one center and one right.

^12^ NEOS (Austria), Social Democrats (Ireland), PDECAT (Spain), Podemos (Spain) and Ciudadanos (Spain).

^13^ NEOS (Austria), Social Democrats (Ireland), Podemos (Spain).

^14^ Only 2 out of 10 left crisis parties, Podemos (Spain) and Social Democrats (Ireland) have higher PIPD values than AIPD. Both score 1 (the maximum) in the PIPD index.

^15^ 51 parties that score 0.5 or more in the digitalization index variable, i.e., that have values manifestly over the mean (0.4).

^16^ AIPD Shapiro–Wilk W = 0.98, p = 0.14; PIPD Shapiro–Wilk W = 0.99, p = 0.84; digitalization Shapiro–Wilk W = 0.99, p = 0.99.

^17^ AIPD and PIPD regression VIF of independent variables < 1.5.

^18^ Parties that score 5 or more in the “populism_cfa_rescaled” variable.

^19^ Intra-party democracy Shapiro–Wilk W = 0.98, p = 0.22.

^20^ Populism Shapiro–Wilk W = 0.91, p = 0.00.

^21^ VIF of all independent variables < 2.
