# Supplementary material for: The internal democracy of the crisis parties in Western Europe: a quantitative analysis of the role of digitalization, ideology and populism
Source: Open Res Eur. 2025 Dec 16;5:288. Originally published 2025 Sep 19. [Version 2] doi: 10.12688/openreseurope.21115.2 (PMC12576320; doi:10.12688/openreseurope.21115.2)
Supplement: Peer review history

# Reviewer report: Reviewer response for version 2

**DOI:** 10.21956/openreseurope.23974.r66219

**Published:** 2025-12-17

**Authors:** Marco Guglielmo

**Affiliations:** 1 University of Valencia, Valencia, Spain

The revised version satisfactorily addresses the suggestions for further improvement.

If applicable, is the statistical analysis and its interpretation appropriate?

I cannot comment. A qualified statistician is required.

Is the study design appropriate and is the work technically sound?

Yes

Is the work clearly and accurately presented and does it engage with the current literature?

Partly

Are the conclusions drawn adequately supported by the results?

Yes

Are sufficient details of methods and analysis provided to allow replication by others?

Yes

Are all the source data and materials underlying the results available?

Yes

Reviewer Expertise:

Political Parties and Digitalization; Ideology-Digitalization Nexus; Political Agency on Digital Platforms; the Politics of Generative Artificial Intelligence

I confirm that I have read this submission and believe that I have an appropriate level of expertise to confirm that it is of an acceptable scientific standard.

# Reviewer report: Reviewer response for version 1

**DOI:** 10.21956/openreseurope.22840.r61260

**Published:** 2025-11-10

**Authors:** Marco Lisi

**Affiliations:** 1 Nova University of Lisbon, Lisbon, Portugal

This paper addresses the important topic of internal democracy in new parties, testing whether newer organizations differ in their degree of intra-party democracy compared to older parties. The study employs comparative datasets and appropriate statistical methods to examine these questions. While the research makes a valuable contribution, several aspects could be strengthened to enhance clarity and analytical rigor.

**Theoretical Framework and Literature Review**

 My first recommendation concerns the presentation of theoretical arguments. The literature review would benefit from greater coherence and structure. Currently, it does not clearly articulate the main analytical dimensions or debates in the field. I suggest reorganizing this section to address the key theoretical themes systematically rather than presenting a fragmented overview. The discussion of new versus old parties should similarly be restructured to provide a more focused analytical narrative rather than dispersed observations.

 I recommend treating the 'theoretical framework' section and the 'hypothesis' section together, as the current separation creates artificial boundaries. The theoretical framework primarily introduces the concept of 'crisis parties' from previous literature rather than developing an integrated analytical framework for the study.

**Hypotheses and Research Design**

 Second, I suggest reformulating Hypothesis 1. The current formulation conflates two distinct propositions: one concerning new parties and another concerning older parties. These should be distinguished as separate hypotheses since they rely on different dependent variables and theoretical mechanisms.

 Third, the operationalization of 'crisis parties' requires further development. Specifically: What electoral threshold is used to classify parties as 'crisis parties'? How does the PPDB dataset criteria align with this conceptualization? A more explicit discussion of case selection and the complete list of parties classified as 'crisis parties' should be provided in the main text or methodological appendix rather than only in supplementary material, to ensure transparency and replicability.

**Presentation of Results**

 Page 14 references Table 3, but the actual table presented is numbered as Table 4. Please verify table numbering throughout the manuscript to ensure consistency.

 Regarding Table 5: I recommend reconsidering the presentation of regression results based on a very small sample (N=39). With such limited observations, the statistical power is questionable and results may be unstable. As an alternative, you might consider adding an interaction term between 'crisis parties' and populism to the main analysis, which would allow you to test the conditional relationship while maintaining adequate sample size. If you retain the separate analysis, please discuss the limitations explicitly and consider reporting measures of model fit and diagnostics to assess reliability.

**Minor Suggestions**

 Overall, the paper tackles a relevant and timely research question with appropriate data and methods. Addressing these structural and analytical refinements will significantly strengthen the contribution and enhance the manuscript's clarity and rigor.

If applicable, is the statistical analysis and its interpretation appropriate?

Yes

Is the study design appropriate and is the work technically sound?

Yes

Is the work clearly and accurately presented and does it engage with the current literature?

Yes

Are the conclusions drawn adequately supported by the results?

Yes

Are sufficient details of methods and analysis provided to allow replication by others?

Yes

Are all the source data and materials underlying the results available?

Yes

Reviewer Expertise:

Political Science

I confirm that I have read this submission and believe that I have an appropriate level of expertise to confirm that it is of an acceptable scientific standard.

# Response

**Published:** 2025-11-28

**Authors:** Jorge Bronet Campos

Dear Marco,

 Thank you for your thoughtful review and helpful comments. They are greatly appreciated. We have reviewed your points, alongside the feedback from the other reviewers. We are currently preparing the revised manuscript and will incorporate your input as we work toward the final resubmission.

 Sincerely,

# Response

**Published:** 2025-12-06

**Authors:** Jorge Bronet Campos

Dear Marco,

 We are pleased to submit the revised version of our manuscript, following the valuable feedback provided by the reviewers.

 We have comprehensively addressed all comments and suggestions. We have incorporated numerous editorial and stylistic changes throughout the text to enhance clarity and analytical rigor, as you recommended. Crucially, we have reorganized the literature review around thematic topics, as suggested. Furthermore, we have integrated our theoretical framework with the reformulated hypotheses (which have been decomposed for greater specificity) to provide a stronger justification for our approach. We have also refined the operationalization of "crisis parties" and eliminated the Table 5. Finally, we have enhanced the concluding section by more explicitly discussing our findings in relation to previous research and clearly delineating their contribution, ensuring the entire text adheres to a consistently formal and appropriate academic tone.

 We believe that these revisions have resulted in a significantly more robust and consistent version of the manuscript. We sincerely hope it now meets your expectations.

 Thank you very much for your time and guidance throughout this process.

 Best regards.

# Reviewer report: Reviewer response for version 1

**DOI:** 10.21956/openreseurope.22840.r61259

**Published:** 2025-10-30

**Authors:** Marco Guglielmo

**Affiliations:** 1 University of Valencia, Valencia, Spain

I am grateful to have the opportunity to review the paper *“The internal democracy of the crisis parties in Western Europe: a quantitative analysis of the role of digitalization, ideology and populism.”* The paper makes a relevant and timely contribution to the fields of party digitalization and intra-party democracy. It innovatively combines the authors’ own generational approach—defining “crisis parties” as a specific cohort emerging between the 2008 economic crisis and the pandemic—with existing large-N surveys (PPDB and POPPA) to quantitatively examine the interplay between digitalization, ideology, and populism in shaping intra-party democracy across Western Europe.

 Overall, I recommend **acceptance with minor revisions**, mainly to enhance clarity, narrative flow, and stylistic consistency. Below I elaborate my comments following the journal’s evaluation criteria.

**Clarity and engagement with the literature**

 The paper is clearly written and empirically grounded, and it engages extensively with two major streams of literature: (1) research on new and digital parties, and (2) studies on intra-party democracy in Western Europe. The review of literature is impressive and comprehensive. Nonetheless, the section *“Internal democracy in new parties of Western Europe: the existing evidence”* could benefit from a more synthetic narrative that connects the various strands of scholarship rather than listing them sequentially.

 A suggested way forward is to guide the reader through a storyline:

- new parties claim to enhance internal democracy through digital tools;
- institutionalization and electoral pressures generate centralization;
- tensions arise between participatory ideals and competitive imperatives.

This structure would highlight the paradox the paper investigates and make clearer how the authors’ quantitative approach contributes to resolving it.

 Stylistically, the authors should avoid colloquial expressions such as “fortunately, data on some of these new parties…”, and ensure consistency in tone. Some signposting would also help, especially to emphasize that the literature simultaneously addresses internal democracy *and* digitalization. For instance, when noting that left-wing parties are more attentive to internal democracy, it could be clarified that the digital parties most often studied (Podemos, La France Insoumise) cluster on that side of the ideological spectrum or at least show distance from right-wing parties (Pirate Parties, Five Stars Movement).

**Theoretical framework**

 The section on *“The generational approach and modes of internal democracy”* is conceptually sound but would benefit from a clearer articulation of its analytical core. Currently, the “generational approach” and the Von dem Berge & Poguntke IPD framework are juxtaposed rather than integrated. The reader would better appreciate the contribution if the authors explicitly stated how the generational approach provides the **population** of cases and the IPD model provides the **analytical lens**.

 This framework should be signposted from the introduction, as it justifies both case selection and variable operationalization. Minor language edits are also needed (e.g., “provides a *good* cohort” → “provides a suitable cohort”; “shed more light” → “shed light”).

**Study design and technical soundness**

 The study design is appropriate, technically solid, and well aligned with the paper’s objectives. The decision to combine PPDB and POPPA data allows for robust cross-validation between organizational indicators and expert assessments.

 However, I would suggest the following adjustments:

- Rename the section *“Hypothesis”* to *“Hypotheses”*, and reformulate them for clarity.
  - **H1a:** Crisis parties—especially left-wing and highly digitalized—are expected to be more plebiscitary than older parties.
  - **H1b:** Older parties—especially on the left—are expected to be more assembly-based.
  - **H2a:** Populism mediates the relationship between crisis-party status and internal democracy.
  - **H2b:** Left-wing populist crisis parties are expected to be more democratic than right-wing populist ones.
    This decomposition would better reflect the paper’s analytical distinctions and facilitate the interpretation of results.
- Some methodological details (e.g., missing cases, coding limitations) could be moved to an appendix, as they currently interrupt the flow of the main text.
- Avoid informal phrasing such as “we bet on the Bronet and Borge’s digitalization index.” Instead, state that the authors *draw on their previously validated digitalization index*.

**Transparency and replicability**

 The data sources are clearly described and publicly available, ensuring replicability. The authors make effective use of open datasets (PPDB and POPPA) and properly acknowledge their limitations. It would nonetheless help to explicitly list the variables included in the models, perhaps in a table, to enhance clarity.

**Statistical analysis and interpretation**

 The statistical procedures (descriptive, bivariate, and bootstrapped OLS regressions) are appropriate and carefully executed. Yet, the interpretation of results would benefit from greater precision. For instance:

- At times, hypotheses are discussed as if they were rejected solely based on descriptive statistics; the logic of inference should be clearly distinguished from descriptive observations.
- The finding that digitalization significantly predicts both AIPD and PIPD should be integrated back into the theoretical argument, highlighting that digitalization—not ideology or cohort—is the key explanatory factor. This insight deserves stronger emphasis throughout the paper.

**Discussion and conclusions**

 The discussion effectively summarizes the findings, but its narrative could be sharpened. The authors convincingly show that:

1. Crisis parties are **not** more plebiscitary than older ones;
2. Digitalization—not cohort or ideology—best predicts intra-party democracy; and
3. Populism negatively affects internal democracy, with left-wing populist crisis parties remaining more democratic than their right-wing counterparts.

This is a valuable contribution that nuances the dominant assumption of digital parties being inherently plebiscitary. However, to strengthen the contribution, I suggest:

- Framing the discussion more explicitly around the **contrast with prior qualitative literature** (e.g., Gerbaudo, Barberà & Rodríguez-Teruel, Deseriis).
- Avoiding vague formulations such as “on a considerable number of political parties,” and instead using “on a large-N sample of Western European parties.”
- Clarifying how the findings advance the understanding of digitalization as a democratizing—or not—force within party organization.

**Style and clarity**

 The manuscript is overall well structured but requires stylistic editing for conciseness and academic tone. Recurrent colloquial expressions (“fortunately,” “we bet on,” “with all these data”) should be replaced with formal equivalents. Some sentences could be simplified for readability, and small grammatical issues (articles, plurals, transitions) should be corrected.

**Final recommendation**

 The article offers a significant and empirically rich contribution to debates on intra-party democracy and digital transformation. It combines innovative conceptualization with robust quantitative evidence. The revisions suggested here are minor and mainly aimed at enhancing readability, narrative coherence, and stylistic precision.

If applicable, is the statistical analysis and its interpretation appropriate?

I cannot comment. A qualified statistician is required.

Is the study design appropriate and is the work technically sound?

Yes

Is the work clearly and accurately presented and does it engage with the current literature?

Partly

Are the conclusions drawn adequately supported by the results?

Yes

Are sufficient details of methods and analysis provided to allow replication by others?

Yes

Are all the source data and materials underlying the results available?

Yes

Reviewer Expertise:

Political Parties and Digitalization; Ideology-Digitalization Nexus; Political Agency on Digital Platforms; the Politics of Generative Artificial Intelligence

I confirm that I have read this submission and believe that I have an appropriate level of expertise to confirm that it is of an acceptable scientific standard.

# Response

**Published:** 2025-11-01

**Authors:** Jorge Bronet Campos

Dear Marco, Thanks so much for your thorough and helpful feedback. We've taken the time to go through all of your comments and those from the other reviewer. We're planning to start revising the manuscript and will certainly use your suggestions to guide our work as we put together the resubmission. Best regards.

# Response

**Published:** 2025-12-06

**Authors:** Jorge Bronet Campos

Dear Marco,

 We are pleased to submit the revised version of our manuscript, incorporating the thorough feedback provided by the three reviewers.

 Following your principal suggestion, we have addressed numerous editorial changes, primarily aimed at enhancing readability, narrative coherence, and stylistic precision. We have made a conscious effort to ensure the text adheres to a more formal and appropriate academic tone throughout.

 In addition to these stylistic revisions, we have implemented several substantive changes: we have restructured the literature review around specific thematic topics and better articulated the purpose and value of our theoretical framework. Furthermore, our hypotheses have been reformulated and decomposed for enhanced clarity. To facilitate fluent reading, some methodological details were relocated to the notes section, and we now more clearly explain our two-step methodology (preliminary analysis and explanatory models). Finally, the conclusions are more explicit and discussed in greater detail in relation to previous research.

 We believe this version is significantly more robust and consistent thanks to your constructive comments. We sincerely hope it now meets your expectations.

 Many thanks for your time and guidance.

 Best regards.

# Reviewer report: Reviewer response for version 1

**DOI:** 10.21956/openreseurope.22840.r61265

**Published:** 2025-10-14

**Authors:** Sorina Soare

**Affiliations:** 1 University of Florence, , Florence, Italy

The paper addresses an important and under-explored question: how intra-party democracy functions within new and electorally successful parties in Western Europe. It builds on well-established frameworks  and introduces an innovative generational approach to analyze crisis parties. The combination of the PPDB and POPPA datasets allows for a valuable large-N comparative perspective. The paper has strong empirical potential and can make a meaningful contribution to the literature on party organization and digital-era democracy.

 That said, a few aspects could be refined to enhance clarity, coherence, and analytical precision.

 The paper rightly notes that existing studies on intra-party democracy in new digital parties are predominantly qualitative and case-based. However, the advantages of adopting a quantitative approach need to be articulated more clearly in the introduction. You could strengthen this point by explicitly explaining how the use of large-N data allows for a systematic testing of the patterns identified in previous qualitative research and what new insights it brings beyond description. The focus on Western parties should also somehow included as an advantage for testing in homogeneous contexts.

 The introduction should define IPD more explicitly, as it is a contested and multidimensional concept. It would be helpful to briefly outline what dimensions of IPD are relevant to your study, participation, deliberation, inclusiveness, and decision-making locus,  and why these were prioritized.

 This clarification would also distinguish between democracy within parties and democracy by parties, avoiding conceptual stretching.

 The theoretical section opens with  an explicit references to left parties. Beginning with a more general reflection on ideological and organizational preferences (rather than directly on left-wing parties) might be beneficial. I also suggest refining phrasing such as “In fact, some new Western European parties…” to “Several new Western European parties especially those on the left have explicitly sought to expand participation and decentralize power.” They look too rough. I also suggest explicitly noting that IPD is not limited to deliberation but also includes decision rules, power distribution, and internal accountability. The end of the literature review should summarize which dimensions of IPD will be tested in the quantitative analysis (e.g., inclusiveness, leader selection, member consultation). This would create a logical bridge between the literature and the empirical design.

 The section on theoretical limitations is valuable but could be structured more clearly. It would help to: explicitly connect the identified gaps (small-N bias, focus on leftist cases, lack of longitudinal perspective) with the advantages of the generational approach; show in a more explicit way how this perspective helps to fill those gaps e.g., by capturing how contextual crises shape party formation and internal organization; clarify how “crisis parties” are operationalized and why this temporal framing is analytically meaningful (also caveats). This would turn a methodological note into a stronger theoretical justification.

 The hypotheses section would benefit from tighter linkage to the theoretical expectations discussed earlier. At present, the rationale for each hypothesis is described, but the causal logic could be made clearer.

 The analytical and discussion sections are strong and well-structured. Still, the paper could benefit from a more explicit dialogue with the initial research gaps identified in the literature review; a more in depth reflection on why crisis parties may not exhibit plebiscitary patterns even when digitalized.

 Beyond these elements, this is a promising and well-crafted paper with clear potential for publication. It combines strong empirical grounding with conceptual ambition. Once the argumentation is streamlined and the theoretical framing slightly reinforced, in my opinion it will stand as a valuable contribution to the comparative study of party organization and intra-party democracy.

If applicable, is the statistical analysis and its interpretation appropriate?

Yes

Is the study design appropriate and is the work technically sound?

Yes

Is the work clearly and accurately presented and does it engage with the current literature?

Yes

Are the conclusions drawn adequately supported by the results?

Yes

Are sufficient details of methods and analysis provided to allow replication by others?

Yes

Are all the source data and materials underlying the results available?

Partly

Reviewer Expertise:

Political aprties, IPD, Digitalisation

I confirm that I have read this submission and believe that I have an appropriate level of expertise to confirm that it is of an acceptable scientific standard.

# Response

**Published:** 2025-11-01

**Authors:** Jorge Bronet Campos

Dear Sorina,

 Many thanks for your valuable feedback. We have carefully reviewed your insightful comments and those from the other reviewer. We will begin working on the revised version of the manuscript, and we will certainly take your points into consideration as we prepare the resubmission. Best regards.

# Response

**Published:** 2025-12-06

**Authors:** Jorge Bronet Campos

Dear Sorina,

 We are pleased to submit a revised version of our manuscript, incorporating the valuable feedback provided by the three reviewers.

 You will notice we have addressed many editorial and stylistic changes. Crucially, as suggested, we have significantly reinforced the argumentation and the theoretical framework. Specifically, we have highlighted the advantages of adopting a quantitative approach, clearly defined the operationalization of IPD, and restructured the literature review around specific thematic topics. Furthermore, we have integrated our theoretical framework with the hypotheses (which have been reformulated) to provide a stronger justification, and made the conclusions more explicit by better discussing them in relation to previous research. We have also made a conscious effort to ensure the text adheres to a more formal and adequate academic tone.

 We believe this version is significantly more robust and consistent thanks to your comments. We sincerely hope it now meets your expectations.

 Thank you very much for your time and guidance.

 Best regards.
